# The anti-angiogenic and cytotoxic effects of the boswellic acid analog BA145 are potentiated by autophagy inhibitors

**DOI:** 10.1186/1476-4598-14-6

**Published:** 2015-01-21

**Authors:** Anup S Pathania, Zahoor A Wani, Santosh K Guru, Suresh Kumar, Shashi Bhushan, Hasan Korkaya, Darren F Seals, Ajay Kumar, Dilip M Mondhe, Zabeer Ahmed, Bal K Chandan, Fayaz Malik

**Affiliations:** Department of Cancer Pharmacology, CSIR-Indian Institute of Integrative Medicine, Canal Road, Jammu, Jammu and Kashmir 180001 India; Academy of Scientific and Innovative Research (AcSIR), New Delhi, 110001 India; Department of Biochemistry and Molecular Biology, Georgia Regents University Cancer Center, 1410 Laney Walker Boulevard CN2136, Augusta, GA 30912 USA; Department of Inflammation Pharmacology, Indian Institute of Integrative Medicine, Canal Road, Jammu, Jammu and Kashmir 180001 India; Department of Biology, Appalachian State University, 572 Rivers Street, Boone, NC 28608 USA

**Keywords:** Autophagy, angiogenesis, hypoxia, vascular endothelial growth factor receptor-2 (VEGFR-2), light chain protein 3 (LC3), BA145, Chloroquinone (CQ)

## Abstract

**Background:**

While angiogenesis inhibitors represent a viable cancer therapy, there is preclinical and clinical data to suggest that many tumors develop resistance to such treatments. Moreover, previous studies have revealed a complex association between autophagy and angiogenesis, and their collective influence on tumorigenesis. Autophagy has been implicated in cytoprotection and tumor promotion, and as such may represent an alternative way of targeting apoptosis-resistant cancer cells. This study explored the anti-cancer agent and boswellic acid analog BA145 as an inducer of autophagy and angiogenesis-mediated cytoprotection of tumor cells.

**Methods:**

Flow cytometry, western blotting, and confocal microscopy were used to investigate the role of BA145 mediated autophagy. ELISA, microvessel sprouting, capillary structure formation, aortic ring and wound healing assays were performed to determine the relationship between BA145 triggered autophagy and angiogenesis. Flow cytometery, western blotting, and microscopy were employed to examine the mechanism of BA145 induced cell death and apoptosis. Live imaging and tumor volume analysis were carried out to evaluate the effect of BA145 triggered autophagy on mouse tumor xenografts.

**Results:**

BA145 induced autophagy in PC-3 cancer cells and HUVECs significantly impeded its negative regulation on cell proliferation, migration, invasion and tube formation. These effects of BA145 induced autophagy were observed under both normoxic and hypoxic conditions. However, inhibition of autophagy using either pharmacological inhibitors or RNA interference enhanced the BA145 mediated death of these cells. Similar observations were noticed with sunitinib, the anti-angiogenic properties of which were significantly enhanced during combination treatments with autophagy inhibitors. In mouse tumor xenografts, co-treatment with chloroquinone and BA145 led to a considerable reduction in tumor burden and angiogenesis compared to BA145 alone.

**Conclusion:**

These studies reveal the essential role of BA145 triggered autophagy in the regulation of angiogenesis and cytoprotection. It also suggests that the combination of the autophagy inhibitors with chemotherapy or anti-angiogenic agents may be an effective therapeutic approach against cancer.

**Electronic supplementary material:**

The online version of this article (doi:10.1186/1476-4598-14-6) contains supplementary material, which is available to authorized users.

## Background

Angiogenesis is the physiological process by which new blood vessels are made from a pre-existing vasculature. While angiogenesis plays an important role in tissue repair and in the formation of the placenta during pregnancy, it is also utilized by malignant cells for their growth and metastasis [1, 2]. Suppressing neoangiogenesis in cancer has been considered an attractive therapeutic option in the era of target based drug discovery, and several anti-angiogenic agents are in clinical development for a number of human malignancies [3, 4]. Despite the anticipated benefits, angiogenesis inhibitors have failed to produce sustained clinical responses in most patients because of resistance towards these inhibitors. It has been proposed that the activation of cytoprotective autophagy accompanies anti-angiogenic drug resistance [5]. Thus, modulating autophagy may enhance the therapeutic activity of anti-angiogenic drugs.

Autophagy removes the unnecessary and dysfunctional cellular components of the cell via a lysosomal degradation pathway. It protects cells and promotes survival during nutrient starvation, infection, or metabolic stress. There is a complex relationship between autophagy and angiogenesis in different types of cancers. Some studies have revealed that autophagy inhibits the angiogenic vasculature [6, 7], while others have suggested that autophagy promotes it [8, 9].

To better understand the cross-talk between angiogenesis and autophagy, we focused on the boswellic acid analog BA145. Our group had recently shown that BA145 is the most potent anti-cancer analogue of boswellic acid, and induces robust apoptosis in human leukemia HL-60 cells [10, 11]. Other studies have reported the anti-metastatic and anti-angiogenic properties of boswellic acids [12, 13]. Here we used a well-studied angiogenesis cell model involving human umbilical vein endothelial cells (HUVECs), the highly aggressive and metastatic human prostate cancer cell line PC-3, as well as mouse models of angiogenesis and cancer to better appreciate the role of autophagy on cell survival, angiogenesis, and tumor progression. Our studies indicate that autophagy plays an important role in cytoprotection by inducing an angiogenic signaling cascade, and that autophagic inhibitors in combination with cytotoxic agents overcome this cytoprotection, thus revealing a previously unknown utility of boswellic acids in cancer therapies.

## Results

### BA145 exhibits antiproliferative and anti-angiogenic properties

**Figure 1:**
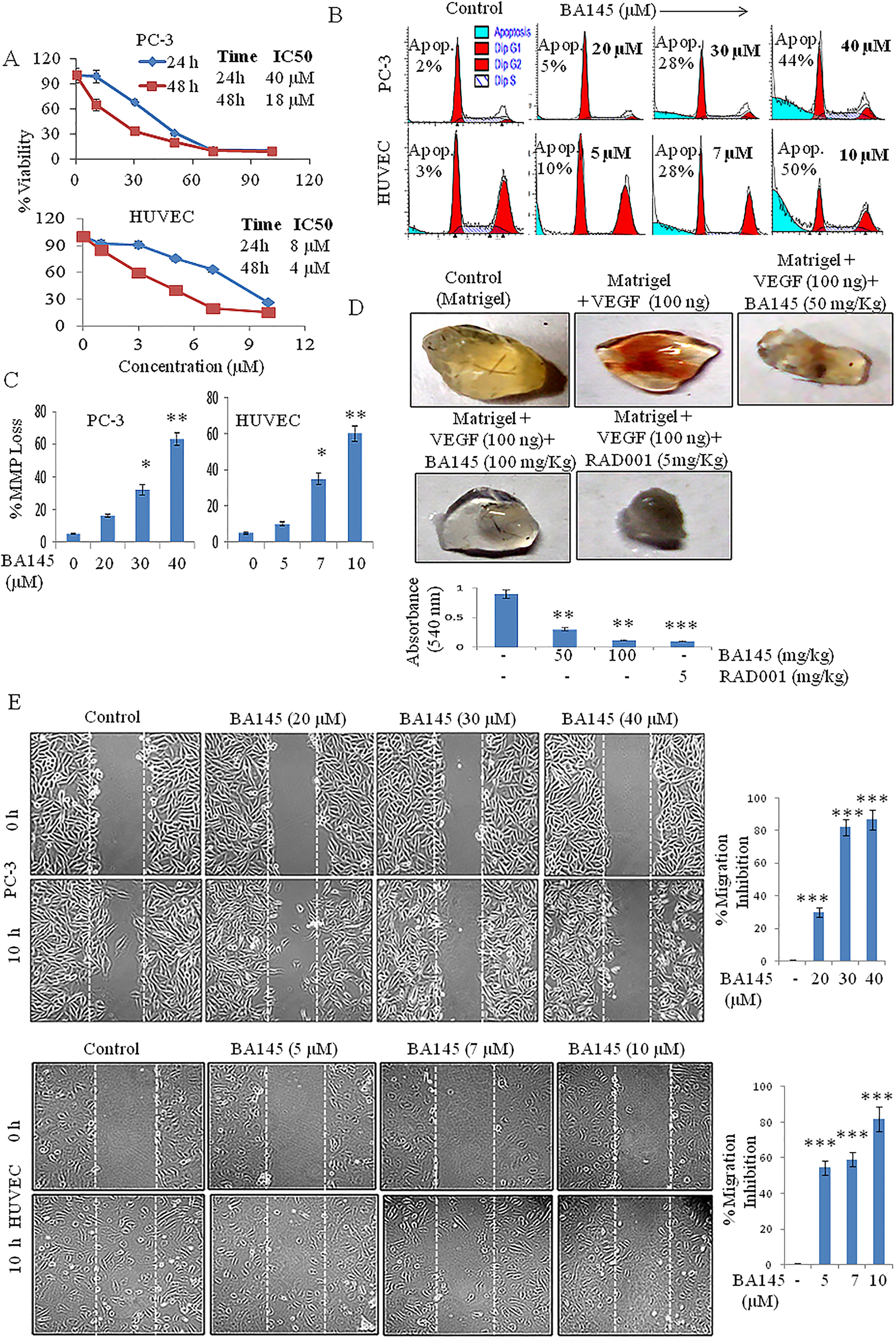
**BA145 exhibits antiproliferative and anti-angiogenic properties. (A)** MTT cell viability assay of PC-3 cells and HUVECs treated with different concentrations of BA145 for 24 and 48 h. **(B)** FACS analysis of SubG1 cell populations following BA145 treatment of PC-3 cells and HUVECs at the indicated concentrations for 24 h. **(C)** Flow cytometric analysis of MMP loss following BA145 treatment of PC-3 cells and HUVECs at the indicated concentrations for 24 h. **(D)** Effect of BA145 on VEGF induced angiogenesis *in vivo.* Six week old C57/BL6J mice were injected with Matrigel containing 50 and 100 mg/kg BA145 along with 100 ng of VEGF into the ventral area. After 10 days, animals were sacrificed to remove Matrigel plugs and were photographed. The neovascularization of the Matrigel plugs were quantified spectrophotometrically using Drabkin’s reagent. **(E)** VEGF induced chemotactic motility in PC-3 cells and HUVECs following treatment with the indicated concentrations of BA145 for 10 h. Migrated cells were counted manually, and the percent inhibition of cell migration was calculated as shown. Columns, mean; bars, SD; with ***p < 0.001, **p < 0.01, *p < 0.05 versus control.

To determine its antiproliferative potential, both PC-3 cells and HUVECs were treated with different concentrations of BA145 for 24 and 48 h. Proliferation was monitored by an MTT assay. BA145 treatment inhibited the proliferation of PC-3 cells with IC50 values of 40 and 18 μM after 24 and 48 h, respectively (Figure 1A). Similarly, treatment of HUVECs with BA145 indicated IC50 values of 8 and 4 μM after 24 and 48 h, respectively. FACS analysis of the BA145 treated PC-3 cells and HUVECs displayed concentration-dependent increases in a SubG1 apoptotic cell population as well as a loss in mitochondrial membrane potential (MMP) (Figures 1B and 1C). The proapoptotic potential of BA145 was further confirmed by observing a dose dependent increase in cleavage of procaspase-3 and PARP-1 in both PC-3 cells and HUVECs (Additional file 1: Figure S1A).

To evaluate the anti-angiogenic potential of BA145 *in vivo,* a Matrigel plug assay was performed in C57/BL6J mice and functional blood vessels were quantified spectrophotometrically by using Drabkin’s reagent. BA145 treatment inhibited VEGF induced blood vessel formation at a dose of 50 and 100 mg/kg when given subcutaneously for 9 days (Figure 1D). RAD001 (5 mg/kg) was used as a positive control. Furthermore, in a wound healing assay it was observed that various concentrations of BA145 inhibited HUVEC and PC-3 cell migration (Figure 1E).

### BA145 inhibits proliferative and angiogenic signaling in PC-3 cells

**Figure 2:**
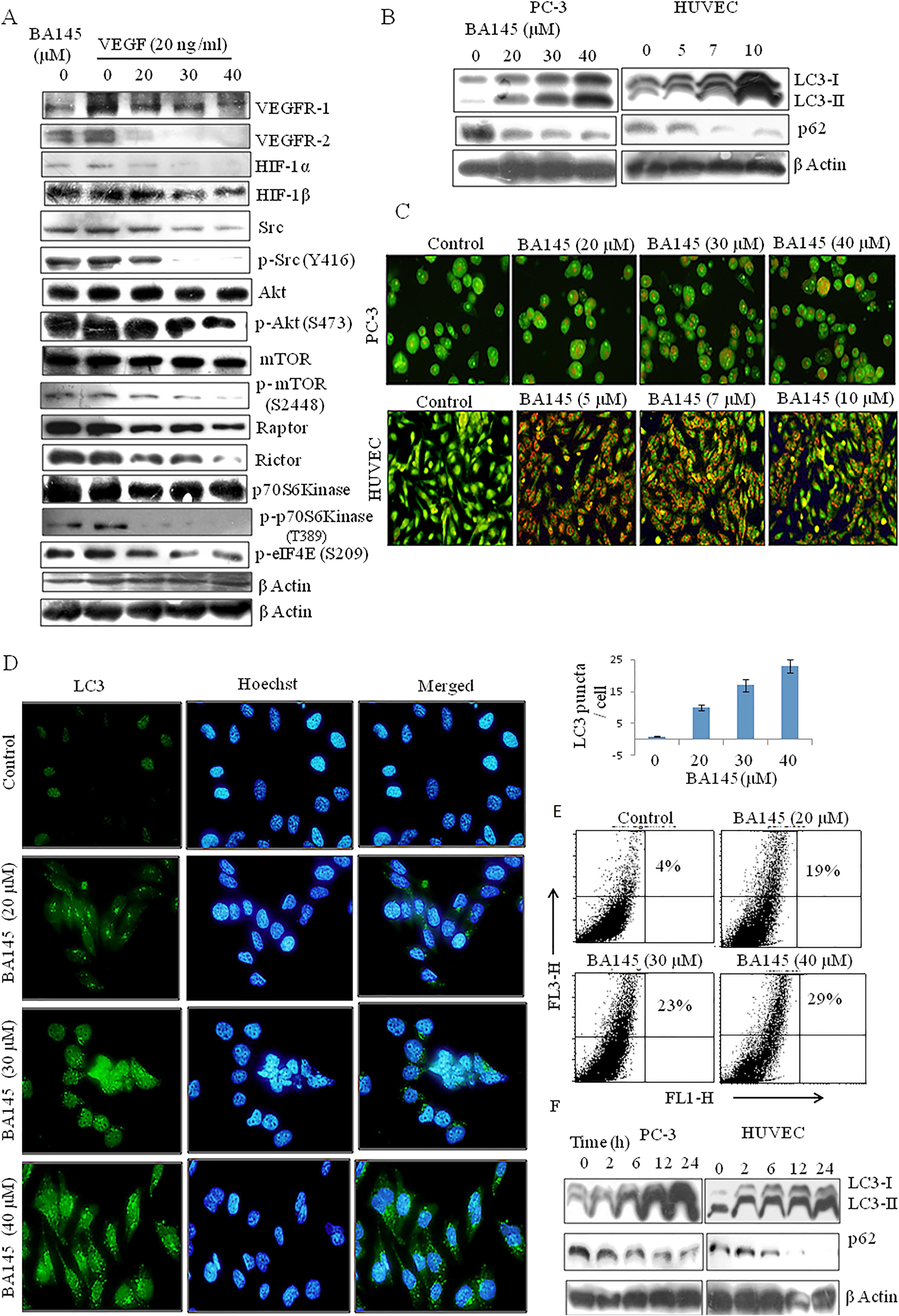
**BA145 triggers autophagy and suppresses VEGFR signaling in cancer cells.** **(A)** Western blot analysis of the indicated proteins in VEGF activated PC-3 cells with or without BA145 treatment for 24 h. **(B)** Western blot analysis of the expression of the autophagy marker proteins LC3 and p62 in BA145 treated PC-3 cells and HUVECs after 24 h. **(C)** Detection of acidic autophagic vesicles in PC-3 cells and HUVECs. After 24 h treatment with BA145, cells were stained with acridine orange (1 μg/ml) in serum free media for 15 min and fluorescent micrographs were obtained by microscopy. Autophagy is indicated by the red fluorescence while the untreated control cells are green. **(D)** Detection of LC3-II protein in PC-3 cells by immunofluorescent microscopy. **(E)** Quantification of acridine orange positive cells by flow cytometry. PC-3 cells were treated with BA145 at the indicated concentrations for 24 h, stained with acridine orange (1 μg/ml) for 15 min, and analyzed with the FL3-H (red color intensity) and FL1-H (green color intensity) channels of the flow cytometer. **(F)** Time dependent accumulation of LC3-II and loss of p62 in BA145 treated PC-3 cells and HUVECs.

VEGF plays a vital role in angiogenesis. VEGF binds to the cell surface receptors VEGFR-1 and VEGFR-2 and activates downstream signaling leading to proliferation, migration, and survival [14]. Hypoxia in tumor tissues induces hypoxia inducible factor-1 (HIF-1) expression, which acts as a transcription factor of genes involved in hypoxic adaptation, promotion of local neovascularisation, and angiogenesis [15, 16]. BA145 treatment significantly inhibited VEGF induced expression of VEGFR-1/R-2 and HIF-1α/1β in PC-3 cells in a dose dependent manner (Figure 2A). Since PI3K/Akt plays a vital role in VEGF mediated angiogenesis [17], we determined whether BA145 was also able to suppress the activation of this signaling pathway. Indeed, treatment of PC-3 cells with BA145 led to downregulation of Akt, Raptor, mTOR, and its downstream substrates p70S6 Kinase and eIF4E (Figure 2A).

### BA145 induces robust autophagy in HUVECs and cancer cells

During autophagy LC3-II is processed from cytosolic LC3-I and expressed on autophagosome membranes along with simultaneous degradation of p62. It was observed that various treatments of BA145 in PC-3 cells and HUVECs for 24 h led to significant increases in LC3-II expression and p62 degradation compared to untreated cells (Figure 2B). Time dependent analysis of BA145 treated PC-3 and HUVECs showed that LC3-II accumulation took place after 2 h along with attendant degradation of p62 (Figure 2F). Acridine orange staining of BA145 treated cells also showed increased formation of acidic vesicles in the cytoplasm (Figure 2C, Additional file 1: Figure S1B). Furthermore, BA145 treatment caused a significant increase in the punctate distribution of LC3-II in PC-3 cells, supporting the notion that LC3-II was localized to autophagososmes (Figure 2D). In PC-3 cells, autophagy initiation by BA145 treatment was confirmed by the increased capture of red fluorescence emitted by the acridine orange dye through flow cytometry (Figure 2E). These experiments collectively demonstrated the BA145 dependent induction of autophagic flux in PC-3 cells and HUVECs. We simultaneously confirmed that BA145 triggers autophagy associated increases in LC3-II expression and acridine orange positive vesicles among the colorectal cancer cell lines HCT116 and COLO205 (Additional file 1: Figures S2A and S2B).

### Inhibition of autophagy accentuates the cytotoxic properties of BA145 and sunitinib

**Figure 3:**
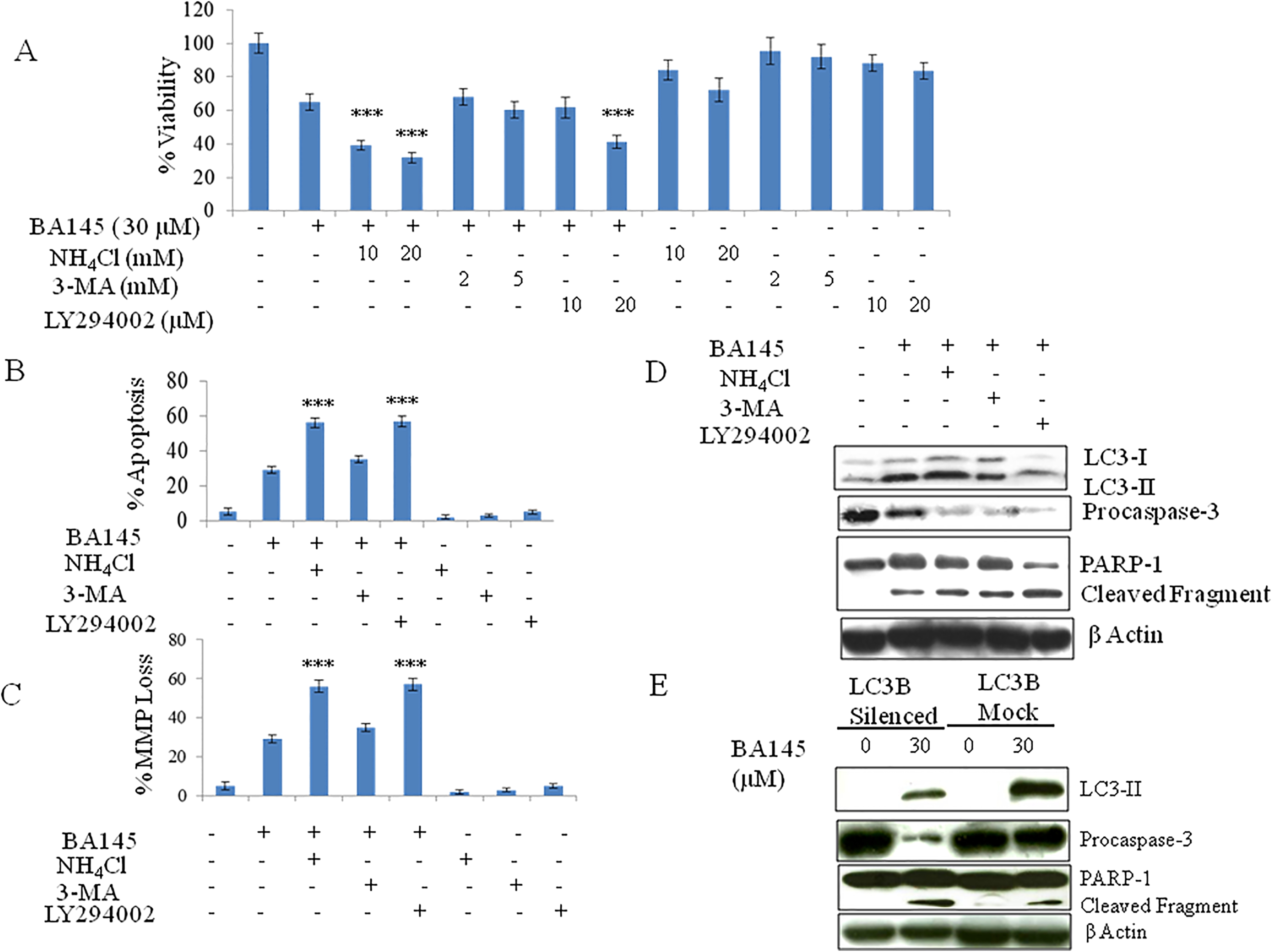
**Effect of autophagic inhibitors on the antiproliferative properties of BA145 in PC-3 cells. (A)** PC-3 cells were co-treated with BA145 (30 μM) and/or the autophagy inhibitors ammonium chloride (10 mM), 3-MA (5 mM), or LY294002 (20 μM) for 24 h. Cell viability was assessed by an MTT assay. **(B, C)** Analysis of SubG1 cell populations and MMP loss in BA145 treated PC-3 cells. Cells were pretreated with autophagy inhibitors for 1 h followed by addition of BA145 (30 μM) for the next 24 h. PI and Rh-123 dyes were used for the analysis of cell cycle and MMP loss, respectively using flow cytometry. **(D)** Effect of autophagic inhibitors on the cleavage of procaspase-3 and PARP-1 in BA145 (30 μM) treated PC-3 cells after 24 h. **(E)** Effect of BA145 on procaspase-3 and PARP-1 cleavage in LC3 silenced cells.

External stimuli or chemically induced stress in tumor cells often triggers autophagy causing cellular dormancy and developmemt of chemoresistance [18]. Inhibitors of such prosurvival autophagy can improve the efficacy of anti-cancer agents when used in combination. BA145 induced autophagy is protective in nature as inhibition of autophagy potentiates BA145 mediated cytotoxicity in PC-3 cells and HUVECs. The combination of BA145 with various autophagy inhibitors, including ammonium chloride (an inhibitor of autophagosome-lysosome fusion) and LY294002 (an inhibitor of the class III PI3kinase activity required for autophagosome formation), significantly enhanced the antiproliferative potential of BA145 in PC-3 cells (Figure 3A) and HUVECs (Additional file 1: Figure S2C) as evidenced by an increase in SubG1 cell populations, MMP loss, and cleavage of procaspase-3 and PARP-1 (Figures 3B, 3C, and 3D, Additional file 1: Figures S2D and S2E). Surprisingly, the combinatorial effect of 3-MA with BA145 did not show any significant alteration on cell survival, though the cleavage of caspase-3 and PARP-1 was considerably increased (Figure 3). To further explore the cytoprotective role of autophagy, we tried to inhibit the expression of key autophagy mediator LC3 in PC-3 cells through siRNA. LC3 silencing promoted the apoptotic effect of BA145 as evidenced by increased procaspase-3 and PARP-1 cleavage when compared to a scrambled siRNA control (Figure 3E). In order to generalize our findings with other anti-angiogenic agents, we treated PC-3 cells and HUVECs with sunitinib. It was observed that sunitinib treatment decreased cell viability, and simultaneously induced autophagy (Additional file 1: Figures S3A, S3B, and S3C). Moreover, inhibition of autophagy with ammonium chloride and bafilomycin greatly enhanced sunitinib induced cytotoxicity. Note that all the pharmacological inhibitors of autophagy were used at noncytotoxic concentrations during these experiments (Figure 3 and Additional file 1: Figure S3).

### BA145 has diverse effects on autophagy among different cancer cell lines

In the colorectal cancer cell lines HCT116 and COLO205, autophagy induced by BA145 is cytoprotective in nature as co-treatment of BA145 with autophagic inhibitors potentiated its cytotoxicity (Additional file 1: Figures S4A and S4B). This was also true in the MCF-7 breast cancer cell line where autophagy inhibition with LY294002 significantly enhanced BA145 mediated cytotoxicity (Additional file 1: Figures S4D and S4E). In contrast, MDA-MB-231 breast cancer cells respond to LY294002 pre-treatment with decreased cytotoxic effects. In the pancreatic cancer cell lines Mia PaCa-2 and PANC-1, co-treatment of BA145 and LY294002 did not show any significant effect on its cytotoxicity. However, LY294002 further enhanced LC3-II accumulation in BA145 treated Mia PaCa-2 cells. Moreover, LY294002 co-treatment in SH-SY5Y neuroblastoma cells was found to enhance the antiproliferative effect of BA145 (Additional file 1: Figures S4D and S4E). Thus, while the impact of BA145 on autophagy can vary among different cancer cell lines, cancers of widely different origins may also respond similarly to BA145 and thereby be favorable targets for combinatorial treatment with autophagic inhibitors.

### Autophagy inhibition potentiates the anti-angiogenic signaling effects of BA145 under both normoxic and hypoxic conditions

**Figure 4:**
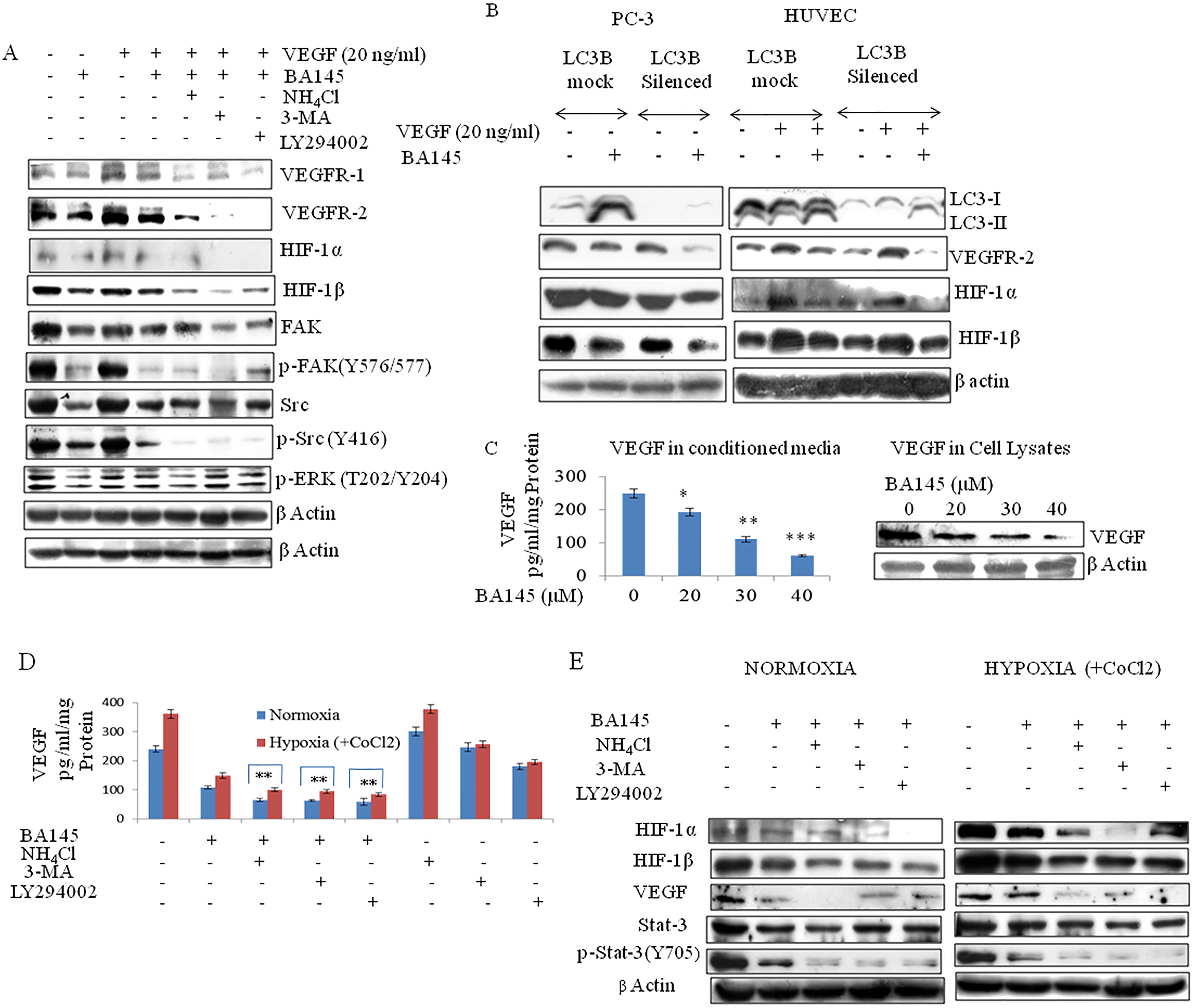
**Autophagy inhibition potentiates the anti-angiogenic effects of BA145.** **(A)** PC-3 cells were treated with BA145 (30 μM) along with autophagic inhibitors for 16 h. Whole cell protein lysates were prepared for western blotting, and the detection of the indicated proteins. **(B)** LC3 specific siRNA were used to silence the expression of LC3 in PC-3 cells and HUVECs that were further treated with BA145 for 12 h. Protein lysates were analyzed by western blotting for the indicated proteins. In the case of HUVECs, prior treatment of VEGF (20 ng/ml) for about 40 min was given before the addition of BA145 (7 μM). For HIF-1α expression in PC-3 cells, VEGF was added 30 min before the BA145 treatment as PC-3 cells have less endogenous HIF-1α expression. **(C)** Effect of BA145 on VEGF production in PC-3 cells. Cells were seeded in 6 well plates, grown to 90% confluency, and then exchanged with fresh media containing BA145 for 16 h. Secreted VEGF in the culture media as well as intracellular VEGF were measured by ELISA and western blot, respectively. Columns, mean; bars, SD; with ***p < 0.001, **p < 0.01, *p < 0.05 versus control. **(D)** Effect of autophagic inhibitors on VEGF production in BA145 treated PC-3 cells under hypoxic and normoxic conditions. Hypoxia was created by treating cells with 100 μM cobalt chloride (CoCl_2_) for 24 h. After incubation, the media was replaced and cells were treated with BA145 (30 μM) and autophagic inhibitors for the next 16 h. Extracellular VEGF in the media was measured by ELISA. Columns, mean; bars, SD; with **p < 0.01 versus BA145 alone. **(E)** Western blot analysis of the indicated proteins following treatment of PC-3 cells with BA145 and autophagy inhibitors under normoxic and hypoxic conditions.

One of the limitations in anti-angiogenic therapies is the creation of hypoxic tumor microenvironments that favor cytoprotective autophagy [19]. To explore the role of autophagy in promoting angiogenesis, we treated PC-3 cells with BA145 along with autophagic inhibitors. The co-treatment of BA145 with ammonium chloride, 3-MA, or LY294002 accentuated the anti-angiogenic properties of BA145 in PC-3 cells. Autophagy inhibitors augmented the inhibition of VEGFR-1/R-2 by BA145, and also augmented the inhibition of downstream signaling molecules like HIF-1α/1β, Src, and FAK (Figure 4A). This was despite the lack of any significant effect of autophagy inhibitors alone on VEGF signaling in PC-3 cells (Additional file 1: Figure S5A). Silencing of the autophagic gene LC3 also accentuated the anti-angiogenic effects of BA145 in PC-3 and HUVECs (Figure 4B). In contrast, the addition of VEGF (20 ng/ml) to BA145 treated PC-3 cells increased the expression of LC3-II as compared to BA145 alone, and this was followed by a decrease in the SubG1 cell population, and in procaspae-3 and PARP-1 cleavage (Additional file 1: Figures S5B, S5C, and S5D).

To generalize our findings with other anti-angiogenic agents, we found that the autophagy triggered by sunitinib treatment of PC-3 cells was accompanied by increased expression of VEGFR-2 and HIF-1α. However, co-treatment of sunitinib with ammonium chloride significantly downregulated the increased expression of these proteins and further potentiated the inhibitory effect of sunitinib on HIF-1β expression as well (Additional file 1: Figure S6).

In order to simulate actual tumor conditions, we next determined the consequences of BA145 mediated autophagy under hypoxic microenvironments. Cobalt chloride was added to the media of PC-3 cells to create hypoxic conditions, and this was followed by treatments with BA145 in combination with inhibitors of autophagy. As shown in Figure 4C, BA145 inhibited cellular and secreted VEGF expression in PC-3 cells. This was further potentiated by co-treatment with autophagy inhibitors (Figure 4D). The switching of normoxic to hypoxic conditions in cancers leads to transcriptional activation of the HIF-1 gene, which is responsible for the induction of genes that adapt cancer cells under oxygen starved conditions [20]. PC-3 cells produce low levels of VEGF and HIF-1α proteins under normoxia, while the expression of these proteins increases under hypoxic conditions (Figure 4E). The treatment of PC-3 cells with BA145 inhibited HIF-1α/1β expression under hypoxic conditions and co-treatment with autophagy inhibitors further augmented this effect. Moreover, the effect of autophagy inhibition was also observed on the expression of Signal transducer and activation of transcription-3 (Stat-3), the constitutive activation of which enhances HIF-1α expression and triggers glycolytic metabolism via the Warburg effect [21, 22]. Our results thus demonstrate that autophagy inhibition in BA145 treated PC-3 cells is able to block this Stat-3 activation under hypoxic conditions (Figure 4E).

### Autophagy inhibition accentuates the inhibitory effects of BA145 on angiogenesis as well as on tumor cell colony formation

**Figure 5:**
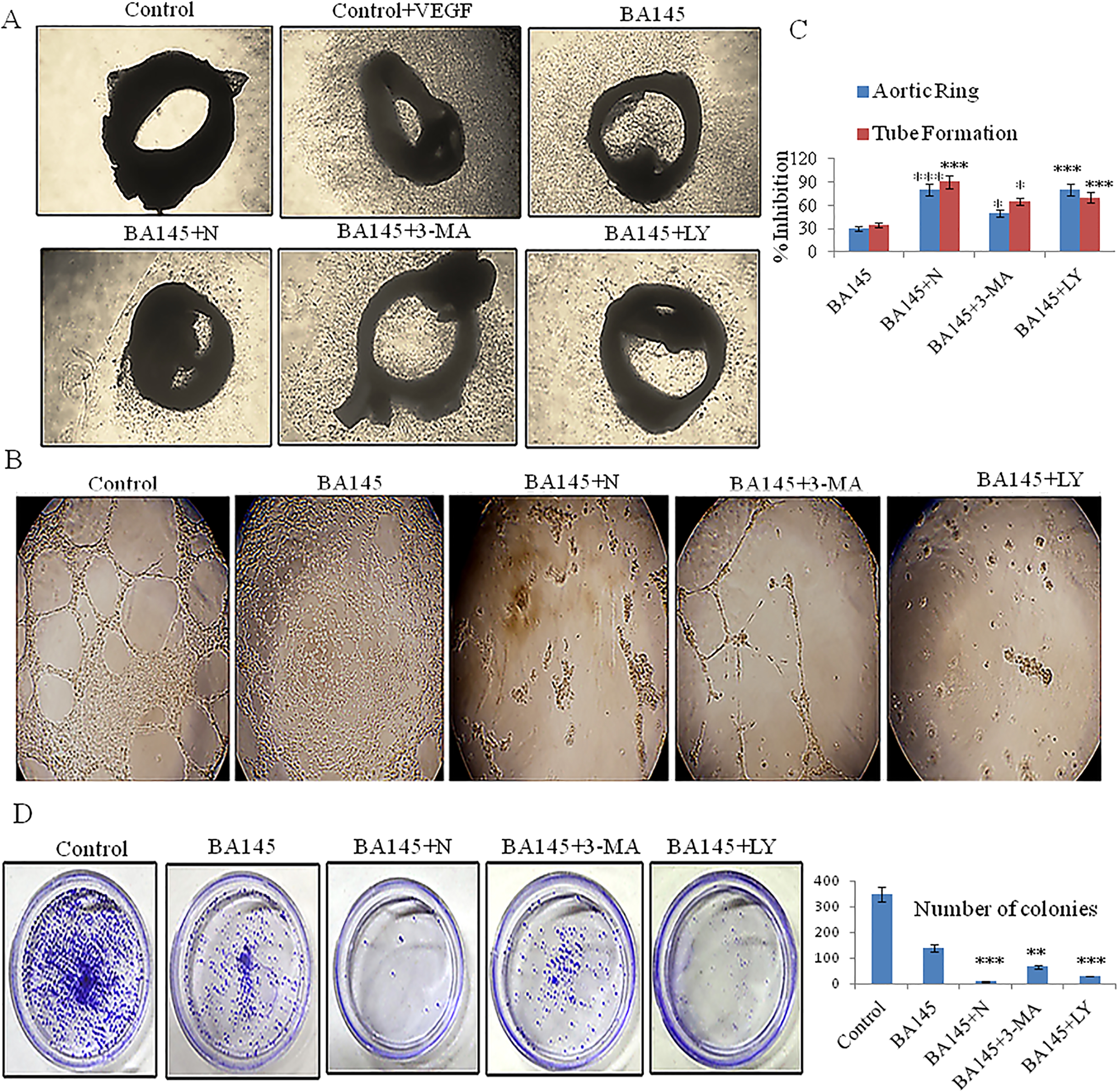
**Autophagy inhibition significantly enhances BA145 mediated anti-angiogenic activity** ***.*** **(A)** Aortic segments were isolated from *Sprague-Dawley rats* and placed in Matrigel-covered wells. BA145 (10 μM) was added to the wells along with ammonium chloride (N; 10 mM), 3-MA (5 mM), or LY294002 (10 μM) for 4 days. Microvessel sprouting was quantitated with Image J software. **(B)** Effect of autophagy inhibition on BA145 mediated suppression of VEGF-induced tube formation. HUVECs were seeded in 24-well plates coated with Matrigel and co-treated with BA145 (10 μM) and autophagy inhibitors for 8 h. Cells were fixed and tubular structures were photographed. **(C)** Percentage inhibition of microvessel sprouting and tube formation of endothelial cells by BA145 along with autophagy inhibitors. **(D)** Colony formation assay of PC-3 cells. Cells were treated with BA145 (30 μM) along with the indicated autophagy inhibitors for 24 h. Cells were trypsinized and 1000 viable cells were seeded in 60 mm dishes. Cells were allowed to form colonies for 15 days, stained with 1% crystal violet, and counted manually. Columns, mean; bars, SD; with ***p < 0.001, **p < 0.01, *p < 0.05 versus BA145 alone.

We used *ex vivo* and *in vitro* models to explore the role of BA145 triggered autophagy in the regulation of angiogenesis. First, we found in an aortic ring assay that autophagy inhibitors enhanced the BA145 mediated suppression of microvessel sprouting (Figures 5A and 5C). Secondly, in a capillary tube formation assay, it was observed that BA145 abolished VEGF induced tube formation by HUVEC cultures and co-treatment with autophagy inhibitors intensified this effect (Figures 5B and 5C). And thirdly, in a wound healing cell assay, it was demonstrated that the effect of BA145 in the inhibition of VEGF induced HUVECs migration was potentiated by autophagy inhibitors (Additional file 1: Figure S7). BA145 treatment also decreased the colony forming ability of PC-3 cells, and the numbers of colonies were significantly decreased during the combinatorial treatment with ammonium chloride, LY294002, or 3-MA (Figure 5D). Importantly, individual treatments with autophagy inhibitors showed no effect on colony formation (Additional file 1: Figure S8A).

### Autophagy inhibition promotes the anti-angiogenic effects of BA145 *in vivo*

**Figure 6:**
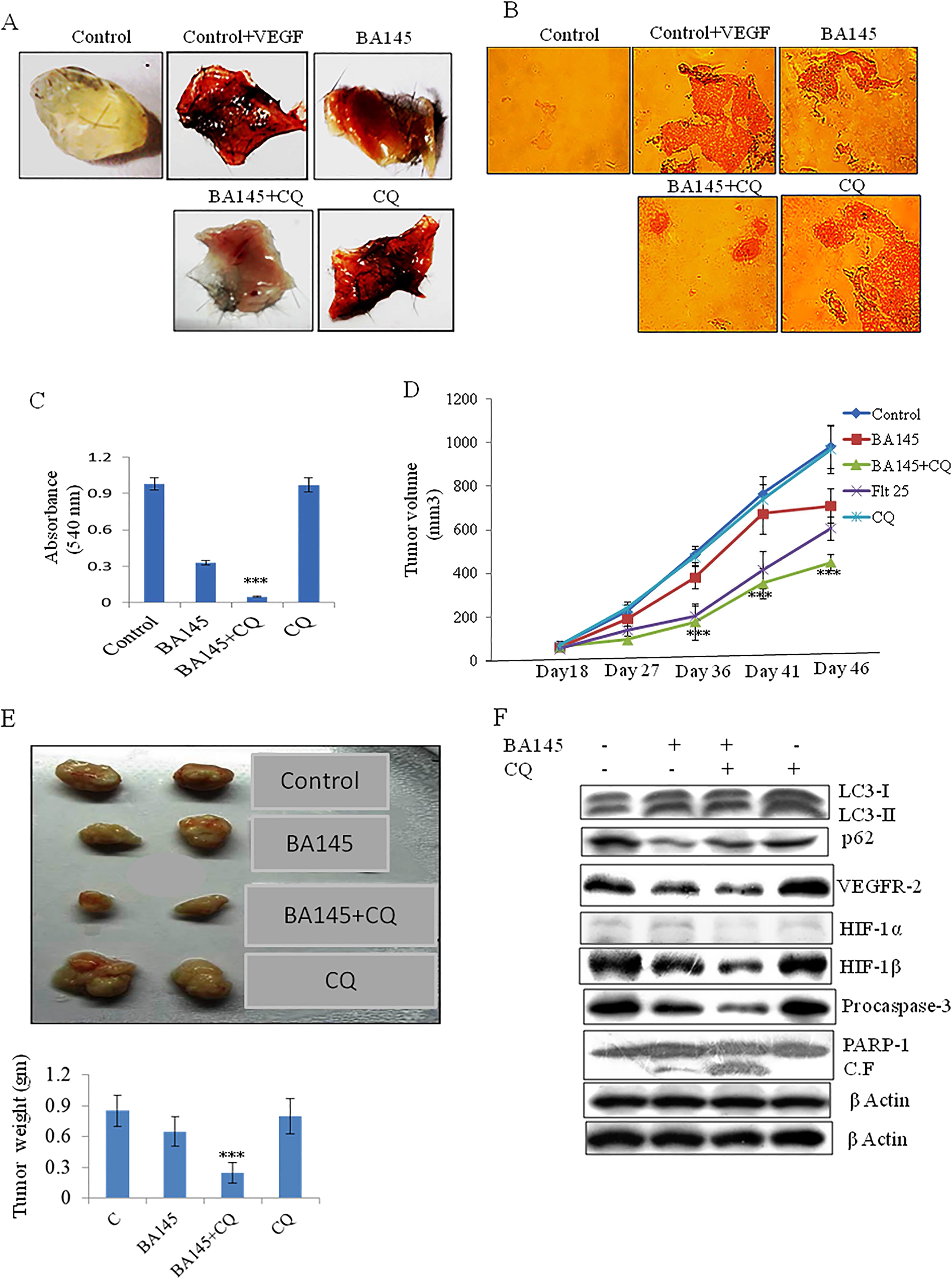
**Autophagy inhibition potentiates the anti-angiogenic effects of BA145** ***in vivo.*** **(A)** Effect of BA145 and CQ on VEGF induced angiogenesis. Six weeks old C57BL/6 J mice were injected with Matrigel containing BA145 (50 mg/kg), CQ (50 mg/kg), or both BA145 and CQ along with VEGF (150 ng) into the ventral area (6 mice per group). Matrigel plugs were monitored 10 days later. **(B)** Matrigel plugs were fixed, sectioned, and stained with H&E. **(C)** Neovascularization of Matrigel plugs were quantified with Drabkin’s reagent using a spectrophometer. **(D)** Effect of BA145 and CQ on tumor growth *in vivo.* PC-3 M-luc2 cells implanted into NOD.SCID mice were treated with vehicle, BA145 (75 mg/kg), BA145 (75 mg/kg) plus CQ (50 mg/kg), positive control flutamide (25 mg/kg), and CQ (50 mg/kg) on alternate days for 28 days. Tumor volumes are reported as mean ± SE. **(E)** Representative pictures of excised tumors and their mass. **(F)** Western blot analysis of the indicated proteins in tumor tissues. β actin was used as loading control. Columns, mean; bars, SD; with ***p < 0.001 versus BA145 alone.

Based on the previously described *in vitro* experiments, we next wanted to determine whether a similar role exists for autophagy inhibitors in BA145 regulation of angiogenesis *in vivo* using the Matrigel plug assay. As shown in Figure 6A, Matrigel plugs containing VEGF are dark red in color due to the formation of a functional vasculature. As expected, treatment with BA145 inhibited angiogenesis, as indicated by the faint color of the Matrigel plugs. Chloroquinone (CQ), an antimalarial drug is known to inhibit autophagy by preventing the acidification of lysosomes, which itself impairs the activity of lysosomal proteases and hence the autophagic degradation process [23]. Co-treatment with CQ and BA145 was able to prominently block Matrigel vasculature formation beyond that of BA145 alone (Figure 6A). H&E staining of the formed vasculature in the Matrigel plugs further confirmed that autophagy inhibition by CQ greatly enhanced the anti-angiogenic activity of BA145 (Figures 6B and C).

### Autophagy inhibitors potentiate the antitumor effect of BA145

Given the success of using CQ to augment the anti-angiogenic properties of BA145, we next determined whether CQ could similarly augment the therapeutic efficacy of BA145 against aggressive PC-3 M-luc2 xenografts in NOD.SCID mice. It was observed that the mean tumor volume increased in control mice from 69.0 ± 18.5 mm^3^ on day 18 to 984.16 ± 100.62 mm^3^ on day 46. This was compared to the 28 day treatment with BA145 that resulted in a tumor volume increase of 60.0 ± 14.68 mm^3^ to 710 ± 77.48 mm^3^ (28% inhibition), and the combination treatment of BA145 and CQ that resulted in a tumor volume increase of 64.0 ± 13.7 mm^3^ to 410.8 ± 36 mm^3^ (58% inhibiton) (Figures 6D and E). Additionally, the average weight of the excised tumors was 0.85 ± 0.15 g in control mice, 0.65 ± 0.14 g in BA145 treated mice, and 0.22 ± 0.1 g in mice treated with both BA145 and CQ (Figure 6E). Interestingly, it was observed that BA145 and CQ co-treatment did not have any significant effect on the body weight of mice (Additional file 1: Figure S8B). Flutamide, an anti-androgen used in the treatment of prostate cancers, was used as a positive control group during the experiment, while CQ at a dose of 50 mg/kg was non-toxic to mice (Figure 6D).

### Inhibition of autophagy enhances the suppression of pro-angiogenesis factors and associated tumor burden in BA145 treated mice

To explore the mechanism by which CQ improved the anti-tumor efficacy of BA145, we examined the changes in angiogenic and apoptotic signals in tumor tissues. Hence, tumor tissues were harvested and lysed in order to monitor the expression of angiogenic and apoptotic factors by western blot analysis. As shown in Figure 6F, BA145 inhibited the activation of VEGFR-2/HIF-1α/HIF-1β in prostate xenografts while the addition of CQ potentiated this effect. Moreover, co-treatment with CQ and BA145 enhanced the cleavage of procaspase-3 and PARP-1 in tumor tissues.

## Discussion

Over the past decade, our understanding of angiogenesis and its role in cancer has increased to a great extent leading to the approval of anti-angiogenic drugs for the treatment of cancer [1, 4]. However, many tumors develop drug resistance with progression of the disease occurring after just a few months of treatment [24, 25]. The molecular mechanism of resistance is not well understood as there are many factors which may play role in this process. Several studies have demonstrated that autophagy plays a crucial role in cell survival and resistance to external stress. Regarding tumors, there are several contentious reports regarding the role of autophagy in angiogenesis and cancer cell death. Although some studies have shown that the autophagy is a different form of cell death [26, 27], others have reported that it is a protective mechanism against chemotherapy related toxicity [28, 29]. In our studies we used the natural compound derivative BA145, a robust autophagy inducer, to investigate the complex association between autophagy, angiogenesis, and cell fate. We are reporting for the first time that BA145 induced autophagy in cancer cells is cytoprotective in nature, and confers protection against the cytotoxic and anti-angiogenic properties of BA145. Using different *in vitro*, *ex vivo* and experimental mouse models, we have shown that BA145 triggered autophagy promotes cytoprotection and angiogenesis. We further show that combinatorial treatments involving BA145 and autophagy inhibitors can be exploited for enhanced tumor cytotoxicity.

BA145 triggered the apoptotic cell death of PC-3 cells and HUVECs based on an observed increase in SubG1 cell populations, a loss in MMP, PARP-1 cleavage, and activation of caspases. BA145 also exhibited anti-angiogenic properties by suppressing VEGF signaling both *in vitro* and *in vivo*. This included the expression of the pro-angiogenic drivers VEGFR-1 and VEGFR-2 as well as several downstream signaling components like HIF-1α, HIF-1β, Src, and FAK. It was further observed that BA145 triggered robust autophagy in PC-3 cells and HUVECs as observed by microscopy, flow cytometry, and the expression of key autophagy proteins. While the role of autophagy in cell death and survival is controversial, our findings conducted in PC-3 cells and HUVECs demonstrated that BA145 induced autophagy is cytoprotective and its inhibition through siRNA or pharmacological inhibitors (e.g. ammonium chloride, 3-MA, or LY294002) augmented the anti-angiogenic and proapoptotic effects of BA145. We further demonstrated that the consequences of BA145 triggered autophagy on cell fate was cell line specific. In the case of HCT116 and COLO205 colon cancer cells, MCF-7 breast cancer cells, and SH-SY5Y neuroblastoma cells, BA145 induced autophagy remained cytoprotective in nature and cytotoxicity was enhanced by autophagy inhibitors. However, in PANC-1 and Mia PaCa-2 cancer cells, autophagy inhibition did not show any significant effect on BA145 mediated cytotoxicity. In MDA-MB-231 breast cancer cells, autophagy inhibition by LY294002 actually decreased BA145 induced cytotoxicity.

Inhibition of autophagy in BA145 treated PC-3 cells enabled significant downregulation of VEGF induced angiogenic signaling compared to BA145 alone. Furthermore, the expression levels of intracellular and extracellular VEGF in PC-3 cells was also diminished. Stat-3 is a point of convergence for many signaling pathways involved in cell proliferation, tumor growth, and angiogenesis [30], and its expression correlates with the expression of VEGF in human cancer cell lines [31]. Activated Stat-3 also mediates the up-regulation of HIF-1α by increasing its stability and transcriptional activity [31]. Interestingly, it was also observed that under conditions of hypoxia, the expression of HIF-1, Stat-3 and VEGF proteins were significantly reduced by combinatorial treatments of BA145 and autophagic inhibitors as compared to BA145 alone. This indicates that autophagy can promote the expression of these pro-survival and pro-angiogenic proteins. The addition of autophagy inhibitors significantly enhanced the anti-angiogenic effects of BA145 as evidenced by reduced capillary like structures, microvessel sprouting and migration in HUVECs. Autophagy inhibition in PC-3 cells enhanced the inhibitory effect of BA145 on colony formation. In mouse models, autophagy inhibition by CQ cooperated with BA145 to completely block vascular formation. And in highly aggressive prostate cancer xenografts in NOD-SCID mice, treatment with BA145 and CQ suppressed tumor growth by 58%, an over 2-fold enhancement relative to BA145 treatment alone. Expression analysis of tumor samples for key angiogenic and autophagy factors revealed that autophagy inhibition by CQ significantly potentiated the negative regulation of BA145 on VEGFR-2, HIF-1α, and HIF-1β, and increased pro-apototic signals. Similarly, inhibiting autophagy in sunitinib treated PC-3 cells and HUVECs greatly enhanced the antiproliferative activity of sunitinib against cancer cells. Consistent with previous studies, it was found that sunitinib treatment increased VEGFR-2 and HIF-1α expression in PC-3 cells [32–34]. This enhanced expression of HIF-1α may be due to the creation of hypoxia, which plays a critical role in the development of angiogenic drug resistance. Our results further demonstrated that the inhibition of autophagy with ammonium chloride in sunitinib treated PC-3 cells led to the supression of angiogenesis as evidenced by the significant decrease in VEGFR-2, HIF-1α, and HIF-1β expression.

## Conclusion

Development of cellular resistance against available anti-cancer or anti-angiogenic agents is a major challenge in the successful treatment of this disease. Understanding the basis of this resistance is critical to the development of novel and effective therapies. During this study, we found that autophagy is one of the mechanisms by which cancer cells protect themselves against cytotoxic or anti-angiogenic agents. Our results showed that the autophagy triggered by the anti-cancer agents BA145 or sunitinib was not only initiated as a survival mechanism, but also promoted angiogenesis. Together these findings suggest that it is important to understand the role of autophagy on cell fate in various cancers triggered by potential anticancer agents. A combination of autophagy inhibitors can be useful in enhancing the therapeutic efficacy of these agents and should be further tested in clinical settings.

## Methods

### Reagents and Antibodies

RPMI-1640, MEM, propidium iodide (PI), rhodamine-123 (Rh-123), acridine orange, 3-(4, 5- dimethylthiazole-2-yl)-2,5 diphenyltetrazolium bromide (MTT), phenylmethanesulfonyl fluoride (PMSF), 3-methyl adenine (3-MA), chloroquinone (CQ), ammonium chloride, bafilomycin, cobalt chloride, sodium fluoride, kanamycin, streptomycin, fetal bovine serum (FBS), β-mercaptoethanol, and human VEGF were purchased from Sigma-Aldrich, Missouri, USA. Growth factor reduced Matrigel were from BD Biosciences, New Jersey, USA. Antibodies for casapse-8, caspase-9, caspase-3, PARP-1, and β-Actin were from Santa Cruz Biotechnology, Texas, USA. All other antibodies and LY294002 were purchased from Cell Signaling Technology, Massachusetts, USA. Electrophoresis reagents, reagents for protein estimation, and protein molecular weight markers were from Bio-Rad Laboratories, California, USA.

### Cell Culture and Treatments

The human prostate cancer cell line PC-3, the colorectal carcinoma cell lines HCT116 and COLO205, the human breast cancer cell lines MCF-7 and MDA-MB-231, the human pancreatic cancer cell lines Panc-1 and Mia PaCa-2, the human neuroblastoma cell line SH-SY5Y and primary human umbilical vein endothelial cells (HUVECs) were purchased from ECACC, England. Cancer cells were grown in RPMI/MEM/McCOY/DMEM growth media containing 10% FBS, 100U penicillin G, and 100 μg streptomycin per ml. HUVECs were grown in EndoGRO-LS complete media from Millipore. Cells were grown in a CO_2_ incubator (Thermocon Electron Corporation, Texas, USA) at 37°C with 95% humidity. BA145, bafilomycin, and LY294002 were dissolved in DMSO (final concentration <0.2%); ammonium chloride, CQ, and 3-MA were solubilized in Milli-Q water.

### Cell Proliferation Assay

Cells were seeded in 96 well plates. When at 70-75% confluency, cells were treated with different concentrations of BA145 for 24 and 48 h. All inhibitors used in the experiment were added 1 h before the treatment of BA145. Cell proliferation was assessed by using an MTT assay as previously described [35].

### Mitochondrial Membrane Potential (MMP) Loss

Cells were treated with or without inhibitors at different concentrations for 24 h. Mitochondrial membrane potential loss (Ψmt) was analyzed by using the fluorescent probe rhodamine 123 as previously described [11].

### Wound Healing Migration Assay

Cells were seeded in 6 well plates. When at 80% confluency, a micro tip was used to draw a scratch across the center of the culture plate to produce a clean wound area. Cells were incubated with BA145 for 8-10 h, and cell migration was examined under the microscope (Olympus Imaging) by manual counting.

### Colony Formation Assay

Cells were treated with BA145 in the presence or absence of autophagic inhibitors at different concentrations for 24 h. Cells were then trypsinized, viable cells were counted, and 1000 cells were seeded in 60 mm dishes for 15 days to determine the effect of these compounds on clonogenic survival. The colonies were fixed in 4% formaldehyde for 15-20 min, stained with 1% crystal violet, and the colonies counted.

### Enzyme-linked Immunosorbent Assay (ELISA) for VEGF

PC-3 cells were seeded in 60 mm dishes. When at 90-95% confluency, cells were treated with BA145 in the presence or absence of autophagic inhibitors and incubated under hypoxic or normoxic conditions for 16 h. Supernatants from the media were collected and the VEGF concentration was determined according to manufacturer guidelines using a VEGF ELISA kit from R&D Systems (#DVE00), Minnesota, USA.

### Capillary-like Tube Formation Assay

A tube formation assay was performed by using an *in vitro* angiogenesis assay kit (Millipore, #ECM 625) according to manufacturer guidelines. Briefly, the extracellular matrix was diluted with diluents buffer before use at 4°C, and 50 μl of this solution was transferred to each well of a pre-cooled 96 well plate. The plate was placed in an incubator at 37°C for at least 1 h to allow the solidification of the matrix solution. HUVECs were trypsinized, and 5000–8000 cells were seeded in each matrix coated well, followed by overnight incubation at 37°C. Cells were treated with BA145 and autophagic inhibitors for 10 h. Tube formation was observed under the light microscope at 10X magnification equipped with a digital camera (Olympus Imaging), and were counted manually.

### Aortic Ring Assay

A rat aortic ring assay was performed as previously described [36]. Briefly, *Sprague Dawley rat*s were obtained from the central animal facility of the institute. Rats were sacrificed by cervical dislocation and aortas were isolated, precleaned from periadventitial fat, and cut into rings at 1 to 1.5 mm in circumference under aseptic conditions. The aortic rings were embedded in Matrigel with or without VEGF (20 ng/ml), followed by treatment with BA145 (10 μM) alone or in combination with LY294002 (10 μM), 3-MA (5 mM) and ammonium chloride (10 mM) for 4 days. Microvessel sprouts were fixed, photographed using an Olympus IX70 inverted microscope, and counted using Image J software [36].

### *In Vivo*Angiogenesis Assay

A Matrigel plug assay was performed as previously described [37]. Briefly, 0.5 ml ice cold Matrigel (BD Biosciences) was injected subcutaneously along with VEGF-A (150 ng/mL), BA145 (50 mg/Kg body weight (BW), and/or CQ (50 mg/kg BW) into C57/BL6J male mice (20-25 g, 4-6 weeks old). After 10 days, mice were sacrificed in order to remove the Matrigel plugs and photographs were taken. The neovascularization of the Matrigel plugs was quantified using Drabkin’s reagent and the absorbance was measured spectrophotometerically at 540 nm in order to estimate hemoglobin (Hb) where Hb (g/dl) = absorbance of sample/absorbance of standard × concentration of standard. All mice were obtained from the central animal facility of the institute. Mice were housed and cared under standard conditions and animal studies were performed according to experimental protocols approved by the Institutional Animal Ethics Committee (IAEC).

### Tumor Studies

The antitumor efficacy of BA145 and the autophagic inhibitor CQ were examined against human prostate cancer xenografts in mice. Male NOD.SCID mice (18-23 g) were procured from the animal facility of the institute and were housed in a sterile microenvironment. The number of animals and the protocols used in this study were approved by the IAEC. PC-3 M-*luc2* cells (3 × 10^6^) were suspended in PBS, mixed with an equal volume of Matrigel, and injected subcutaneously into the right flanks of each animal on day 0. Tumor growth was monitored on every alternate day and animals bearing 50-100 mg tumor mass were selected for the experiment on the staging day (day 18). Mice were randomly distributed into 5 different groups with 6 mice in each group. Group I was administered 0.2 ml normal saline (i.p.), and served as an untreated control. Group II was treated with BA145 (75 mg/kg i.p.) on every alternate day. Group III was administered BA145 (75 mg/kg i.p.) and CQ (50 mg/kg i.p.) on every alternate day. Group IV was administered flutamide (25 mg/kg p.o.), and served as a positive control. Group V was administered with CQ (50 mg/kg i.p.) on every alternate day. Tumors volumes were calculated as described earlier [38]. On day 46, all animals were sacrificed and tumors were excised. Cell lysates were prepared from the harvested tumors using RIPA buffer for western blotting.

### Immunofluorescence and Confocal Microscopy

PC-3 cells were grown on coverslips and treated with BA145 at different concentrations for 24 h. Cells were fixed, stained, and analyzed under the fluorescent microscope as previously described [11].

### Preparation of Cell Lysates and Western Blot Analysis

The preparation of cell lysates and protein specific immunoblotting was performed as previously described [11].

### Statistical Analysis

Data are presented as means of three similar experiments and the error bars represent the standard deviation (SD) between the experiments. Statistical analysis was done using the Bonferroni method and a p value <0.05 was considered to be significant (***p < 0.001, **p < 0.01, *p < 0.05).

## Electronic supplementary material

Additional file 1: Figure S1: BA145 induced apoptosis and autophagy in PC-3 and HUVECs. **Figure S2**: Role of BA145 induced autophagy in cancer cell lines. **Figure S3**: Inhibition of autophagy enhanced sunitinib mediated cytotoxicity in PC-3 cells and HUVECs. **Figure S4**: Effect of autophagy inhibitors on BA145 mediated cytotoxicity in cancer cell lines. **Figure S5** (A) Effect of ammonium chloride (10 mM), 3-MA (5 mM), or LY294002 (20 μM) on angiogenic signaling proteins in PC-3 cells. Cells were treated with these inhibitors for 16 h and protein lysates were prepared for western blot analysis of the indicated proteins. (B) VEGF addition increases LC3-II expression in BA145 treated PC-3 cells. Cells were treated with BA145 (30 μM) in the presence or absence of VEGF (20 ng/ml) for 24 h. Lysates were prepared and western blotting of the indicated proteins was performed. (C and D) MTT assay and SubG1 cell cycle analysis of BA145 treated PC-3 cells in the presence or absence of VEGF. Columns, mean; bars, SD; with *p < 0.05 versus BA145 alone. **Figure S6**: Effect of ammonium chloride on VEGFR-2, HIF-1α and HIF-1β expression in sunitinib treated PC-3 cells. **Figure S7**: Combinatorial effects of BA145 and autophagy inhibitors on VEGF induced chemotaxis of endothelial cells. **Figure S8** (A) Colony formation in ammonium chloride (10 mM), 3-MA (5 mM), or LY294002 (20 μM) treated PC-3 cells after 24 h. Cells were trypsinized and 1000 viable cells were seeded in 60 mm dishes. Cells were allowed to form colonies for 15 days after which colonies were stained with 1% crystal violet and photographed. (B) Body weight changes in mice treated with BA145, CQ, and/or flutamide (Flt25). There was no significant difference in body weight between the treated groups and the control group in this study. (DOC 19 MB)

## Abbreviations

AO: acridine orange
LC3: microtubule-associated protein light chain 3
MTT: 3-(4,5-Dimethylthiazole-2-yl)-2,5-diphenyltetrazolium bromide
PI: propidium iodide
Rh-123: Rhodamine-123
CoCl_2_: Cobalt chloride
siRNA: small interfering RNA
HUVECs: human umbilical cord vein endothelial cells
VEGFR: vascular endothelial growth factor receptor
CQ: Chloroquinone
BA145: Boswellic acid analogue.
