# Supplementary material for: The anti-angiogenic and cytotoxic effects of the boswellic acid analog BA145 are potentiated by autophagy inhibitors
Source: Mol Cancer. 2015 Jan 21;14:6. doi: 10.1186/1476-4598-14-6 (PMC4509694; doi:10.1186/1476-4598-14-6)
Supplement: Supplementary file 1 — Additional file 1: Figure S1: BA145 induced apoptosis and autophagy in PC-3 and HUVECs. Figure S2: Role of BA145 induced autophagy in cancer cell lines. Figure S3: Inhibition of autophagy enhanced sunitinib mediated cytotoxicity in PC-3 cells and HUVECs. Figure S4: Effect of autophagy inhibitors on BA145 mediated cytotoxicity in cancer cell lines. Figure S5 (A) Effect of ammonium chloride (10 mM), 3-MA (5 mM), or LY294002 (20 μM) on angiogenic signaling proteins in PC-3 cells. Cells were treated with these inhibitors for 16 h and protein lysates were prepared for western blot analysis of the indicated proteins. (B) VEGF addition increases LC3-II expression in BA145 treated PC-3 cells. Cells were treated with BA145 (30 μM) in the presence or absence of VEGF (20 ng/ml) for 24 h. Lysates were prepared and western blotting of the indicated proteins was performed. (C and D) MTT assay and SubG1 cell cycle analysis of BA145 treated PC-3 cells in the presence or absence of VEGF. Columns, mean; bars, SD; with *p < 0.05 versus BA145 alone. Figure S6: Effect of ammonium chloride on VEGFR-2, HIF-1α and HIF-1β expression in sunitinib treated PC-3 cells. Figure S7: Combinatorial effects of BA145 and autophagy inhibitors on VEGF induced chemotaxis of endothelial cells. Figure S8 (A) Colony formation in ammonium chloride (10 mM), 3-MA (5 mM), or LY294002 (20 μM) treated PC-3 cells after 24 h. Cells were trypsinized and 1000 viable cells were seeded in 60 mm dishes. Cells were allowed to form colonies for 15 days after which colonies were stained with 1% crystal violet and photographed. (B) Body weight changes in mice treated with BA145, CQ, and/or flutamide (Flt25). There was no significant difference in body weight between the treated groups and the control group in this study. (DOC 19 MB) [file 12943_2014_1497_MOESM1_ESM.doc]

SUPPLEMENTARY DATA


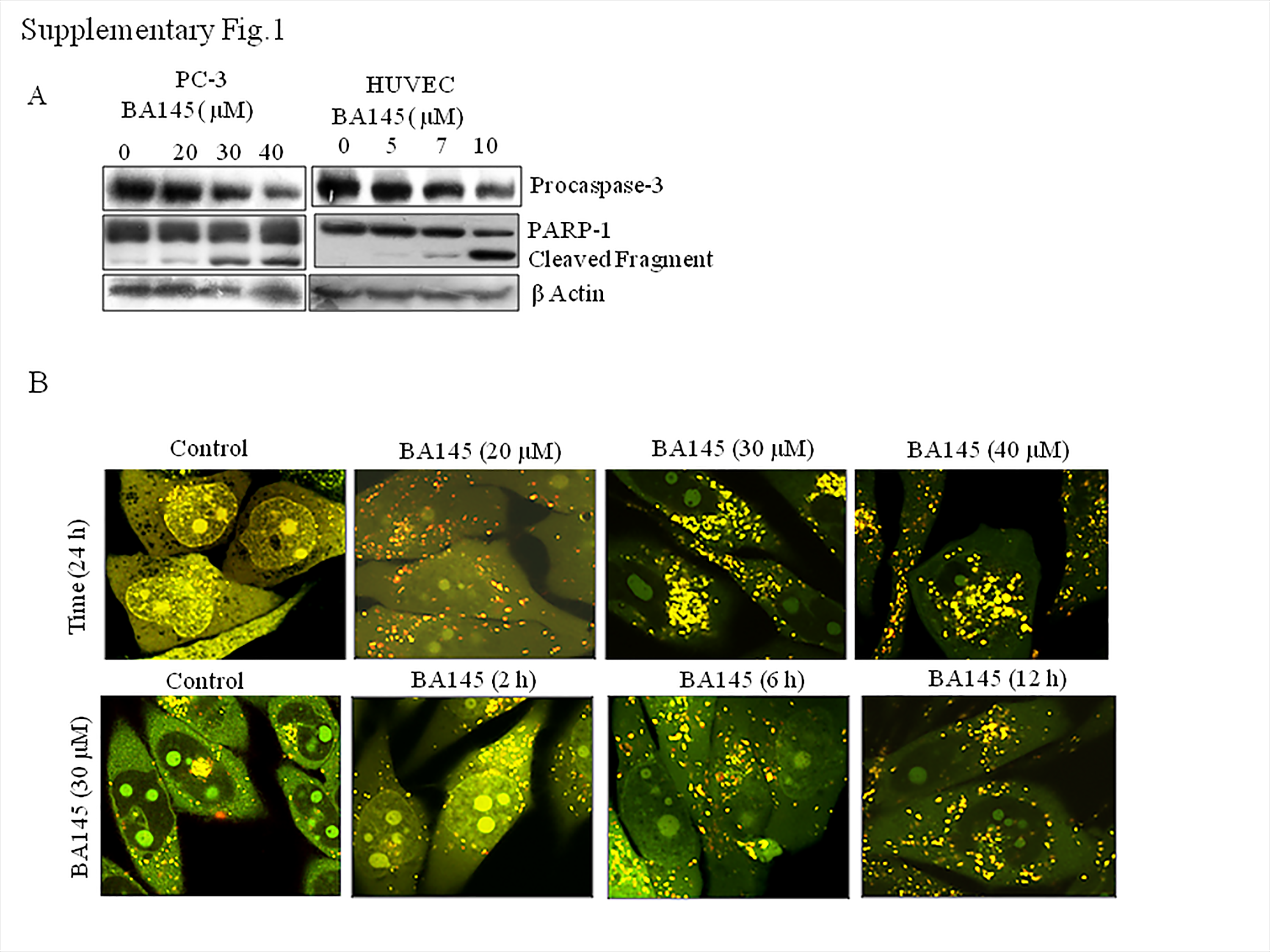


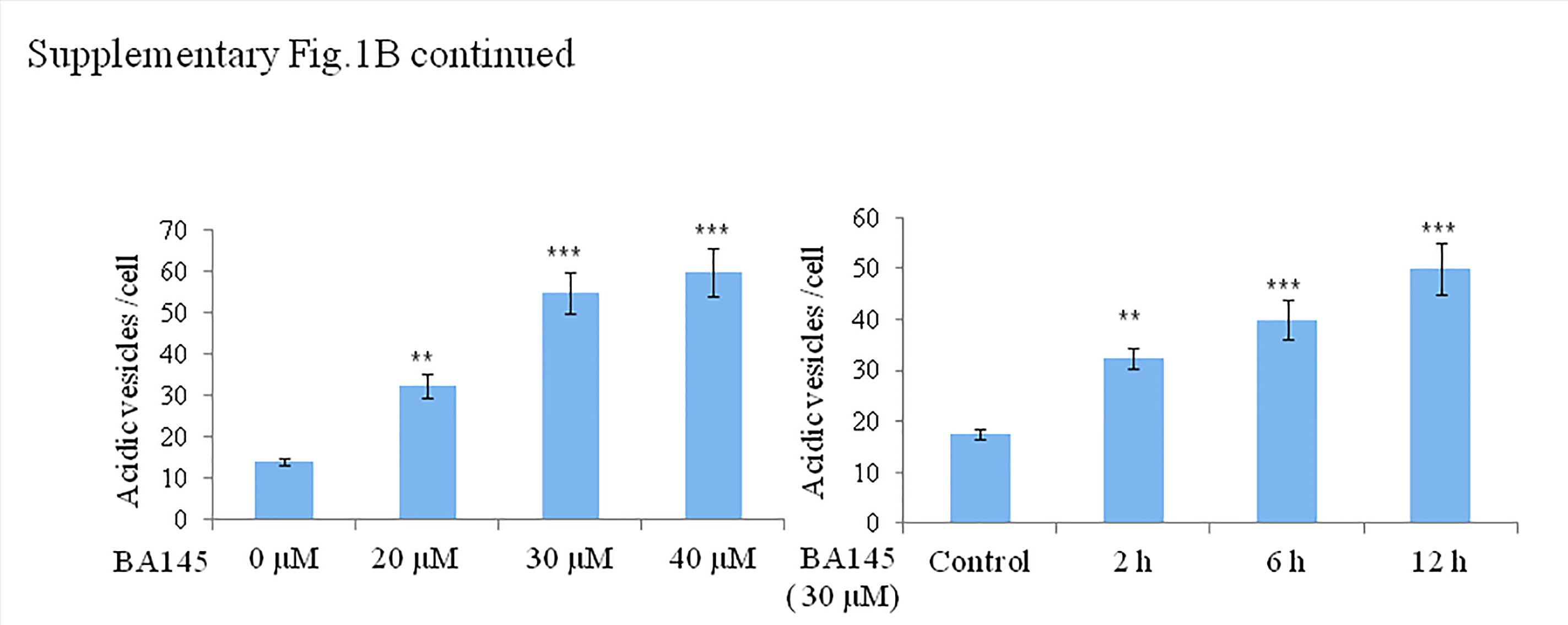


**Figure S1. BA145 induced apoptosis and autophagy in PC-3 and HUVECs.** (**A**) Cells were treated with BA145 at the indicated concentrations for 24 h, then harvested for preparation of whole cell protein lysates and subsequent western blot analysis for Procaspase-3, PARP-1, and β-actin. (**B)** BA145 triggered robust acridine orange positive, acidic vesicle formation in PC-3 cells in a time and concentration dependent manner as analyzed by confocal fluorescence microscopy. Columns, mean; bars, SD; with ***p<0.001, **p<0.01 versus control.


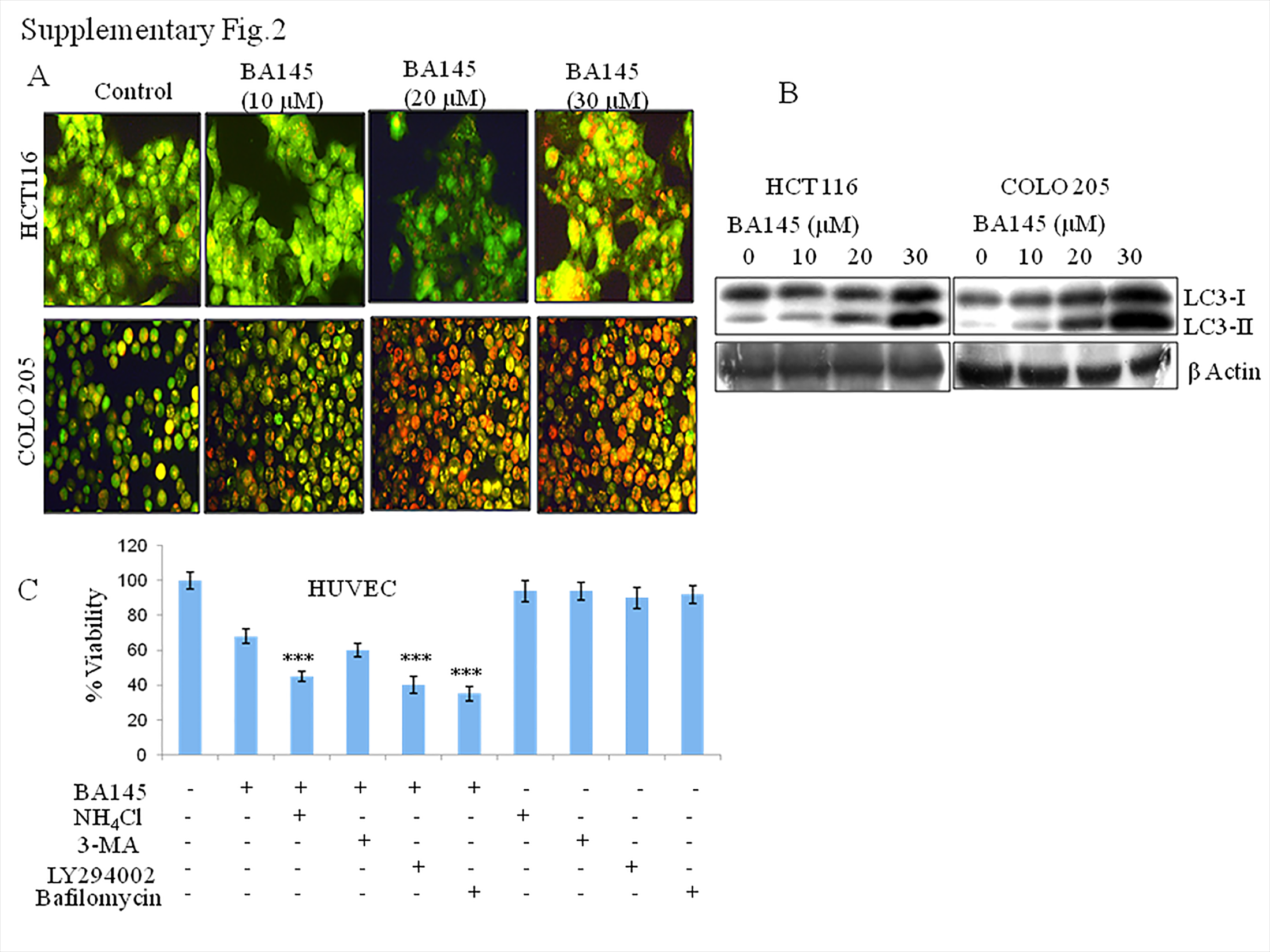


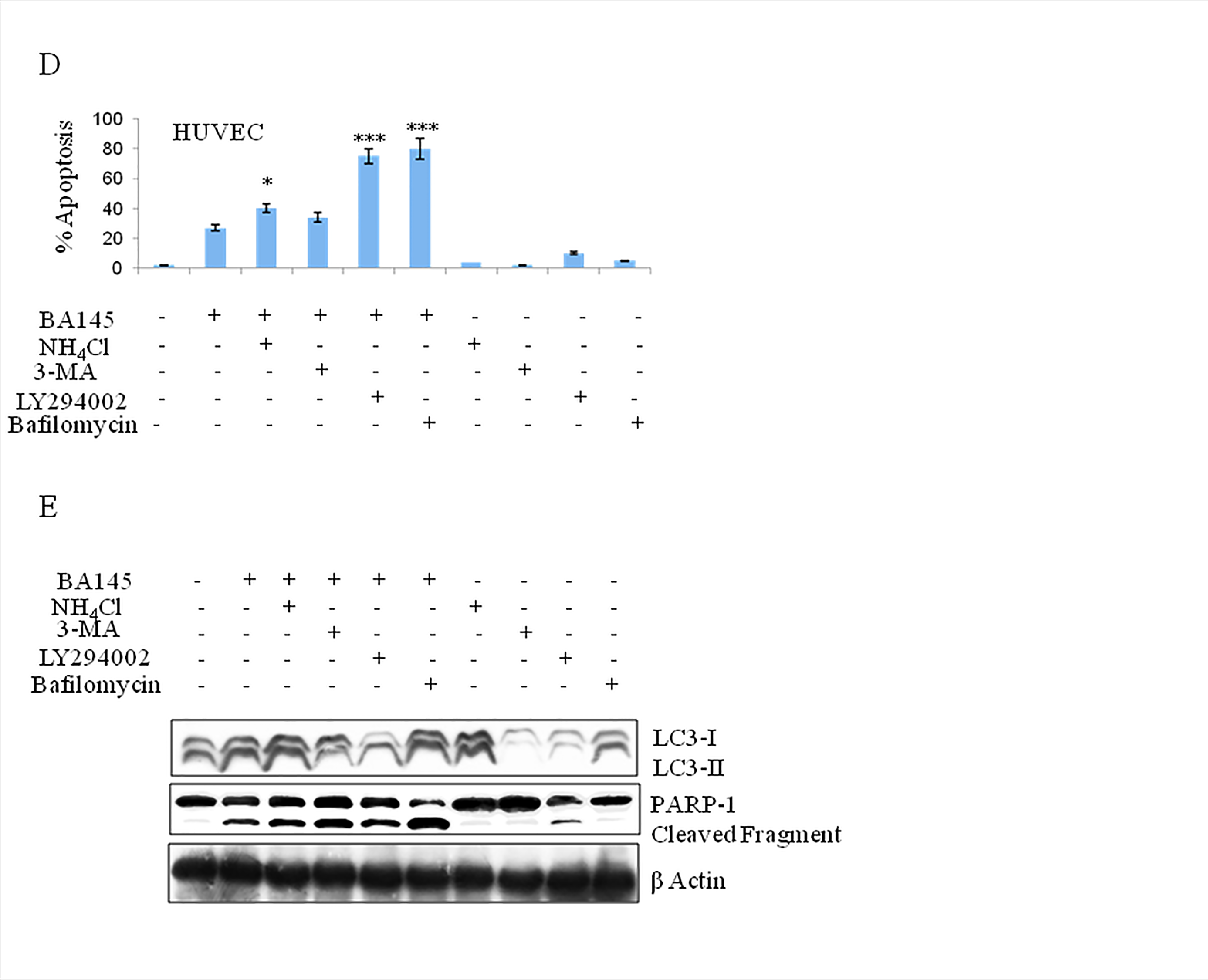


**Figure S2. Role of BA145 induced autophagy in cancer cell lines. (A**) BA145 induced protective autophagy in the colon cancer cell lines HCT116 and COLO205. Cells were treated with BA145 at the indicated concentrations for 24 h. Cells were stained with acridine orange for 15 min and visualized by confocal fluorescence microscopy. (**B**) BA145 increased LC3-II levels in HCT 116 and COLO 205. Cells were treated with BA145 at the indicated concentrations for 24 h. Lysates were prepared for western blotting of LC3 protein. (**C**) Effect of autophagy inhibitors on the BA145 mediated cytotoxicity in HUVECs. Cells were treated with BA145 (7 µM) and the autophagy inhibitors ammonium chloride (10 mM), 3-MA (5 mM), LY294002 (10 µM), or bafilomycin (10 nM) for 24 h. Cell viability was calculated using an MTT assay. All inhibitors were added 1 h before BA145 treatment. (**D**) Cell cycle analysis of HUVECs treated with BA145 and autophagy inhibitors as described in panel C. Cells were fixed, stained with PI, and the percentage of apoptotic cells was determined by flow cytometry. Cell cycle data were analyzed by using ModFit software. **(E)** Pharmacological inhibition of autophagy enhanced PARP-1 cleavage in BA145 treated HUVECs. After the compounds treatments described in panel C, cells were harvested for whole cell protein lysate preparation and subsequent western blot analysis for LC3, PARP-1, and β-actin. Columns, mean; bars, SD; with ***p<0.001, *p<0.05 versus BA145 alone.


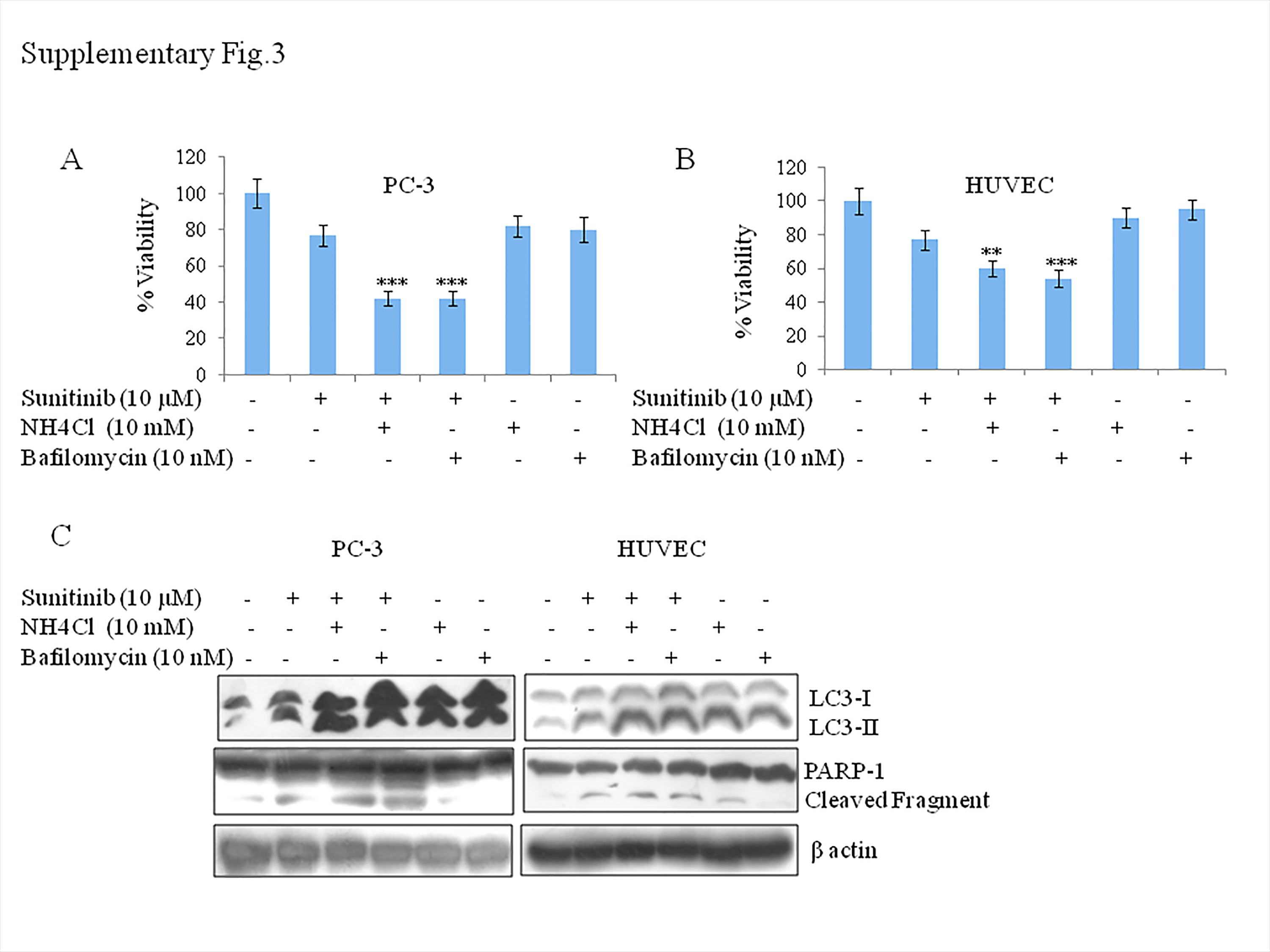


**Figure S3. Inhibition of autophagy enhanced sunitinib mediated cytotoxicity in PC-3 cells and HUVECs. (A, B)** Cells were treated with sunitinib (10 µM) along with the indicated autophagy inhibitors for 24 h. Cell viability was calculated by an MTT assay. **(C)** Effect of autophagy inhibitors on LC3 expression and PARP-1 cleavage in sunitinib treated PC-3 cells and HUVECs. Columns, mean; bars, SD; with ***p<0.001, **p<0.01 versus sunitinib alone.


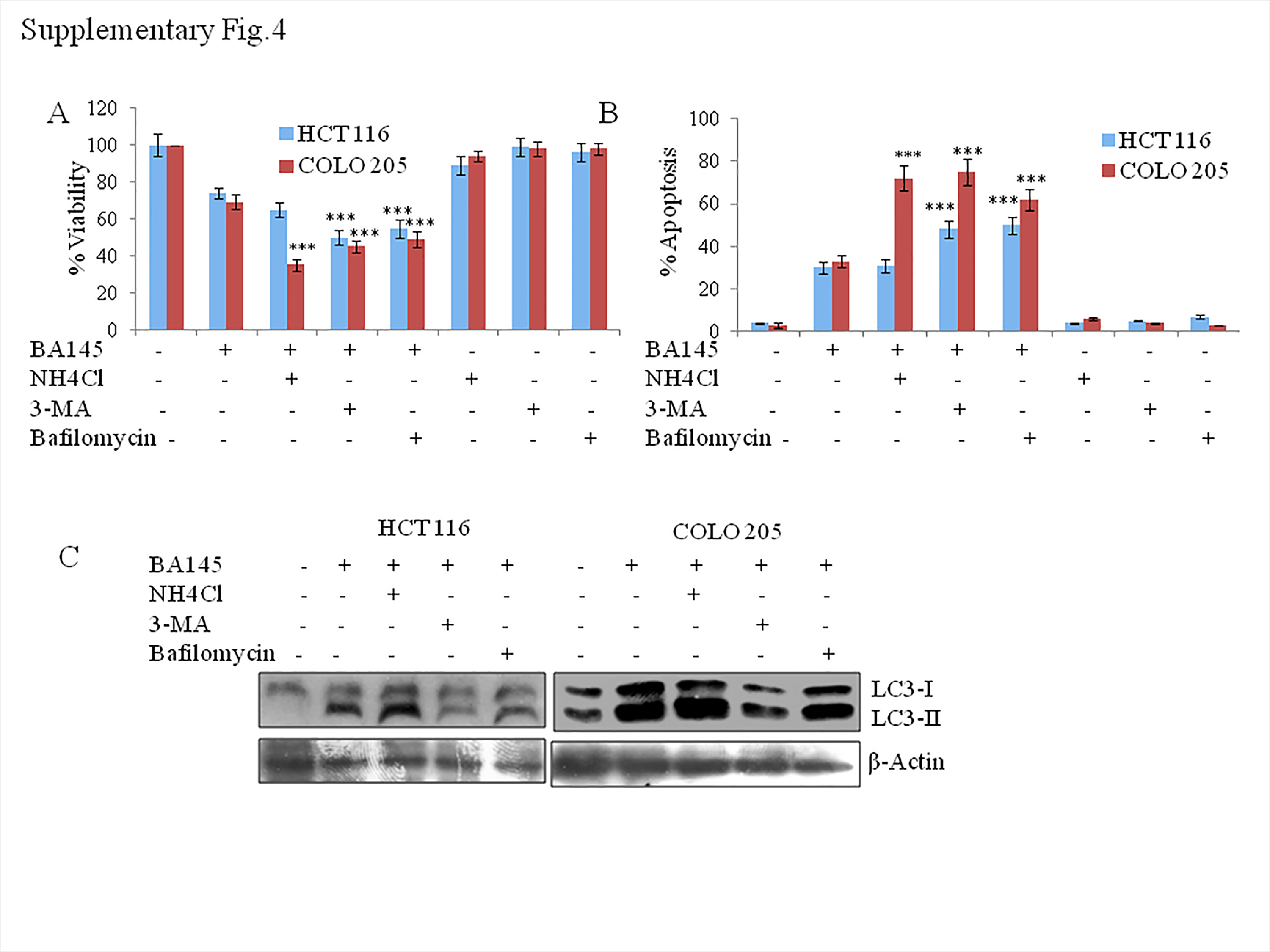


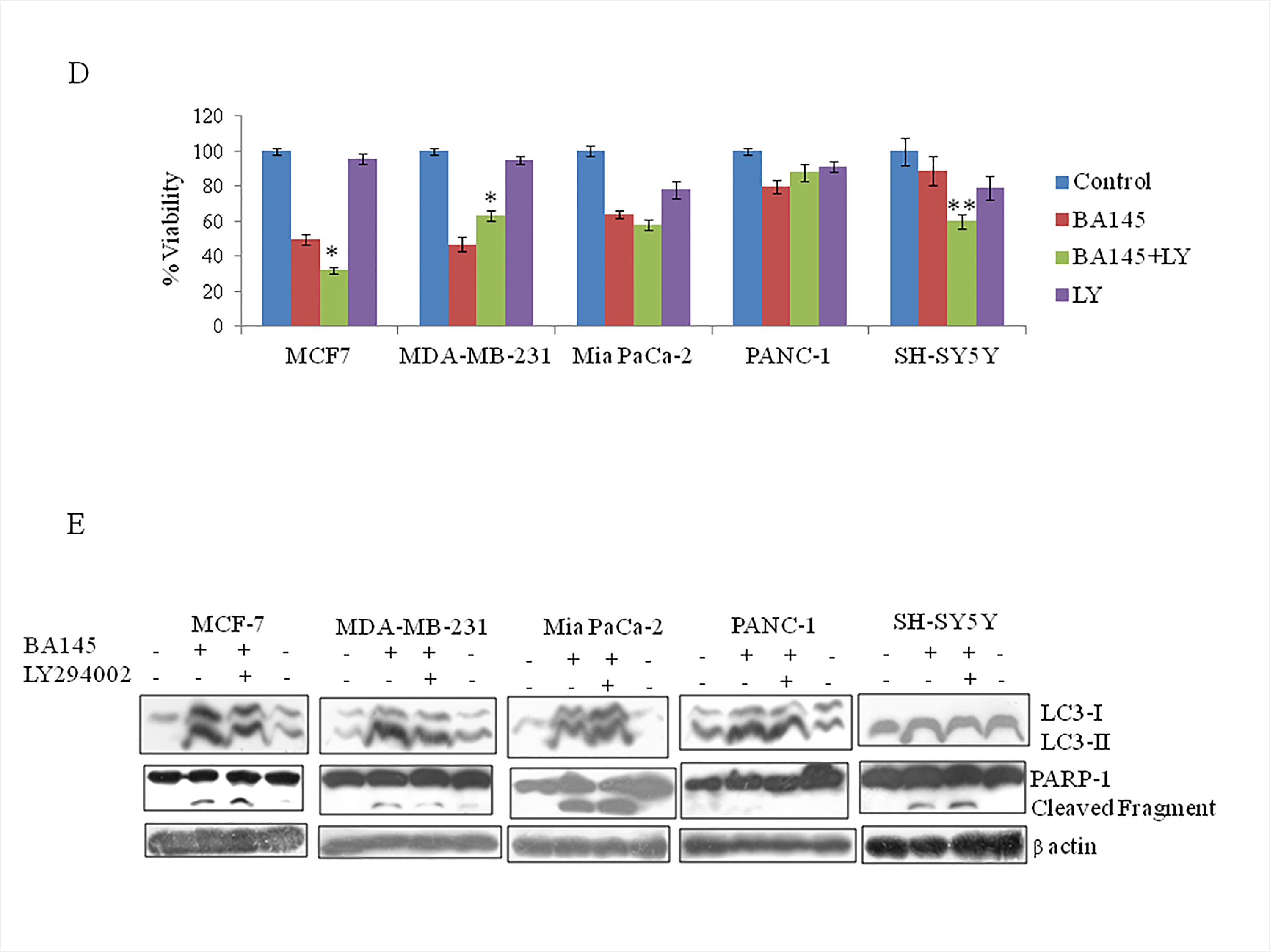


**Figure S4 Effect of autophagy inhibitors on BA145 mediated cytotoxicity in cancer cell lines**. (**A**) Autophagy inhibitors enhanced BA145 mediated cytotoxicity in colon cancer cell lines. Cells were treated with BA145 (30 µM) alone or in combination with ammonium chloride (10 mM), 3-MA (5 mM), or bafilomycin (25 nM) for 24 h. Cell viability was estimated by using an MTT assay. (**B**) SubG1 cell cycle analysis of cells co-treated with BA145 and the indicated autophagy inhibitors for 24 h. (**C**) Effect of BA145 and autophagy inhibitors on LC3 expression in HCT116 and COLO205 cells. Columns, mean; bars, SD; with ***p<0.001, versus BA145 alone. **(D** and **E)** Effect of the autophagy inhibitor LY294002 on BA145 treated cancer cell lines. The breast cancer cell lines MCF-7 and MDA-MB-231, and the pancreatic cancer cell lines Mia PaCa-2 and Panc-1 were treated with 40 µM BA145 for 24 h, while the neuroblastoma cell line SH-SY5Y were treated with 10 µM BA145 for 24 h. LY294002 (20 µM) was added 1 h before BA145 treatment. Cell viability was calculated by an MTT assay and LC3-II accumulation and PARP-1 cleavage were analyzed by western blotting. Columns, mean; bars, SD; with **p<0.01, *p<0.05 versus BA145 alone.


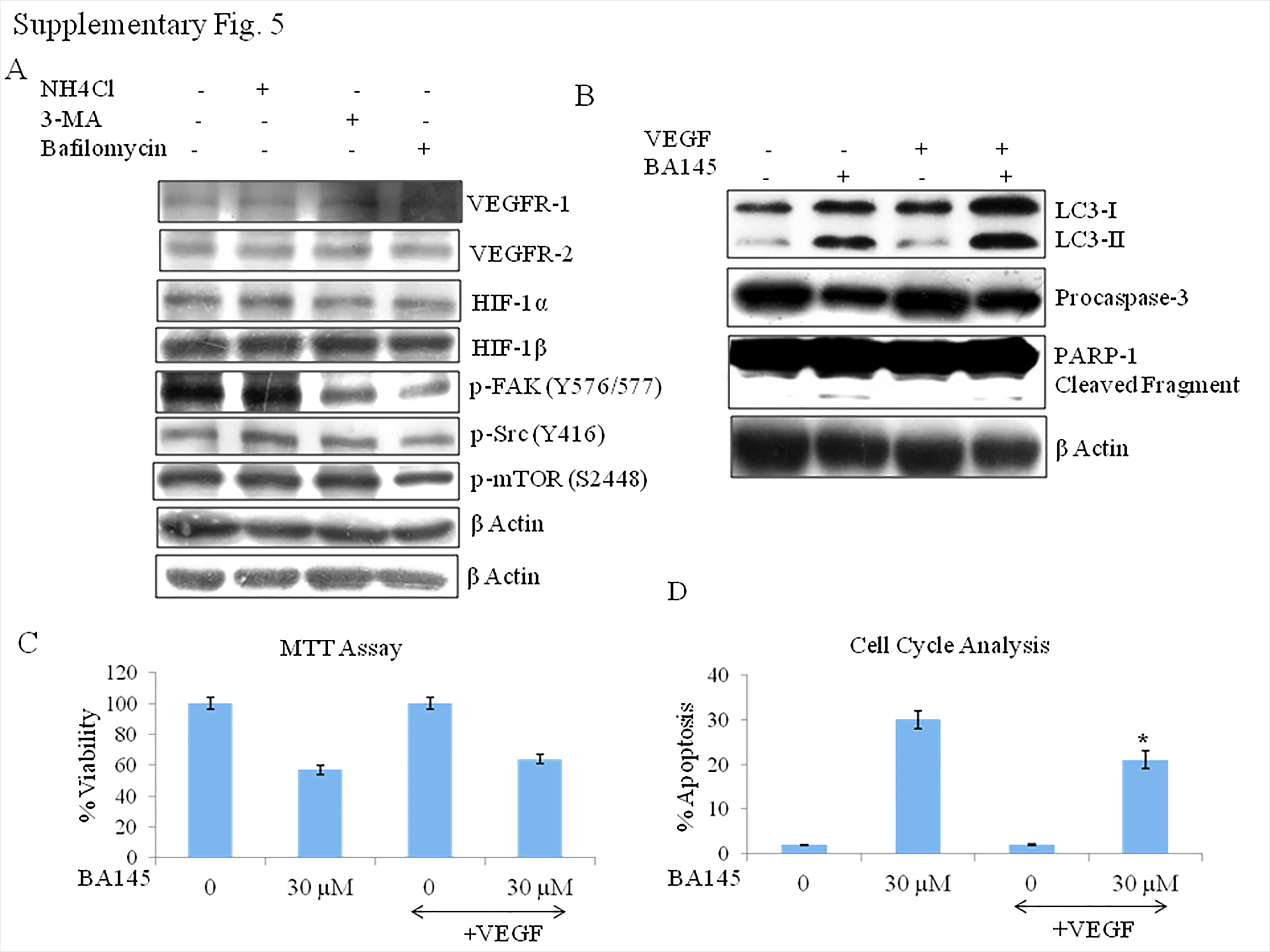


**Figure S5 (A**) Effect of ammonium chloride (10 mM), 3-MA (5 mM), or LY294002 (20 µM) on angiogenic signaling proteins in PC-3 cells. Cells were treated with these inhibitors for 16 h and protein lysates were prepared for western blot analysis of the indicated proteins. (**B**) VEGF addition increases LC3-II expression in BA145 treated PC-3 cells. Cells were treated with BA145 (30 µM) in the presence or absence of VEGF (20 ng/ml) for 24 h. Lysates were prepared and western blotting of the indicated proteins was performed. (**C** and **D**) MTT assay and SubG1 cell cycle analysis of BA145 treated PC-3 cells in the presence or absence of VEGF. Columns, mean; bars, SD; with *p<0.05 versus BA145 alone.


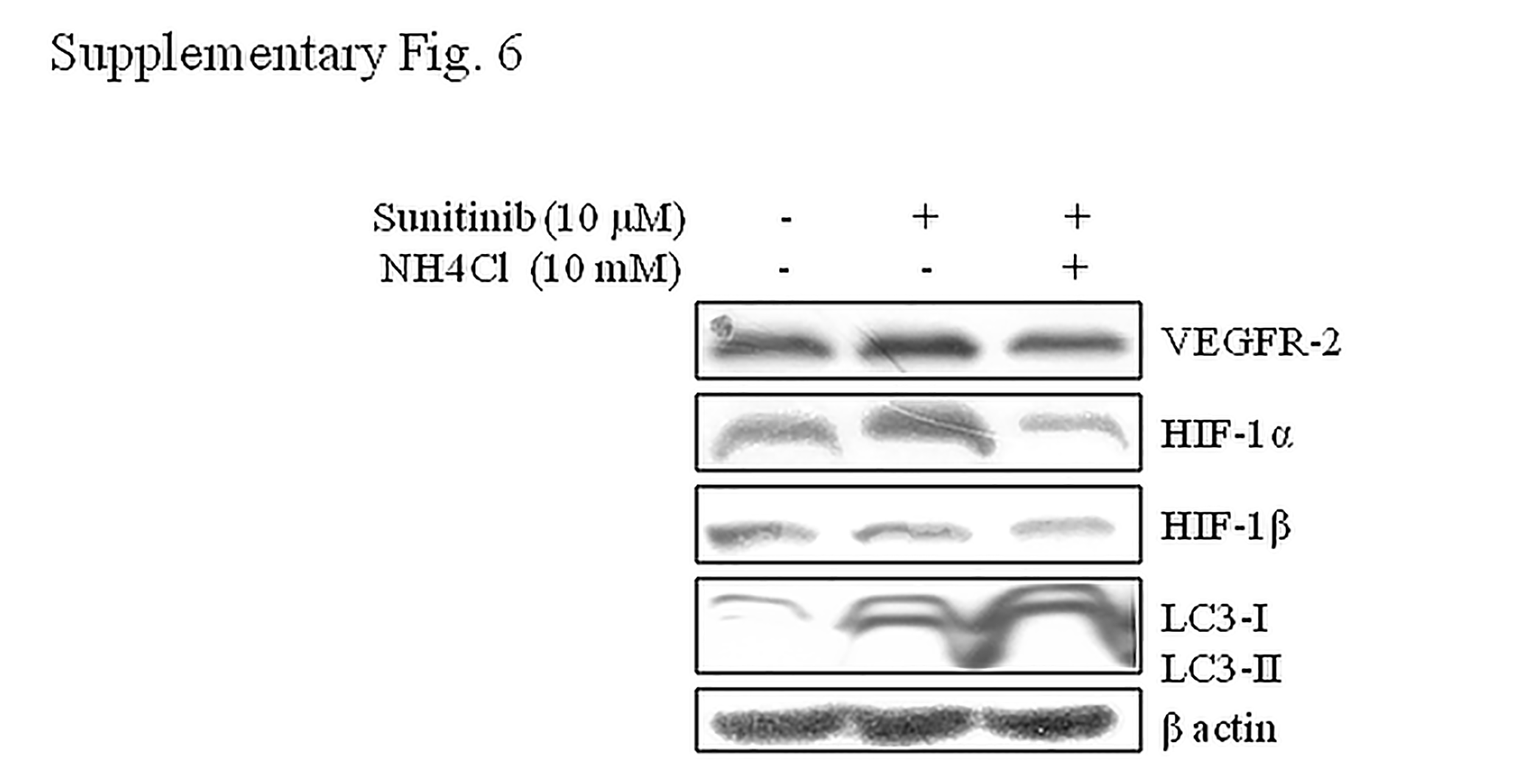


**Figure S6. Effect of ammonium chloride on VEGFR-2, HIF-1α and HIF-1β expression in sunitinib treated PC-3 cells**. Cells were treated with sunitinub (10 µM) and ammonium chloride (10 mM) for 10 h time period. Lysates were prepared and expression of the indicated proteins was examined.

**
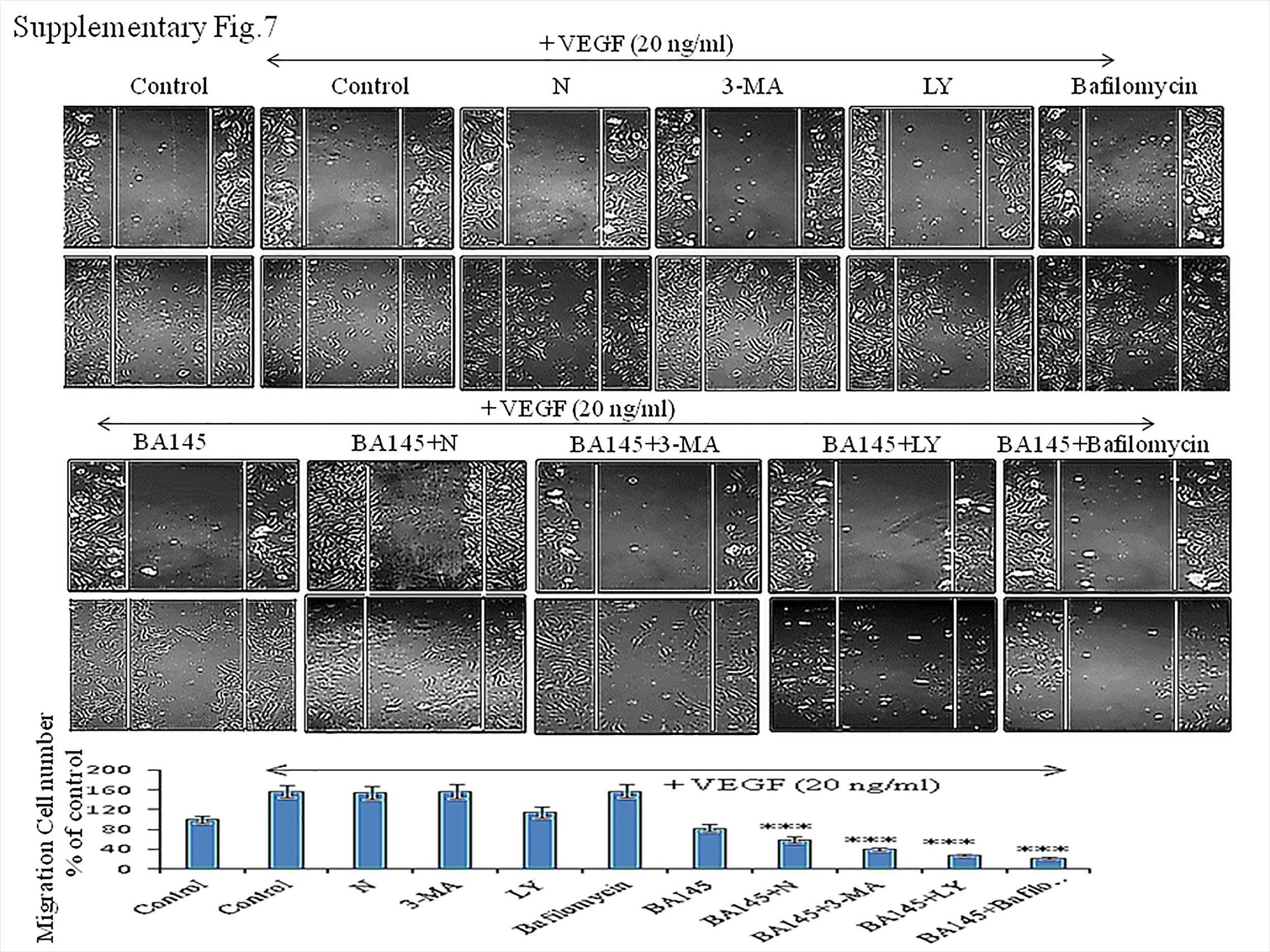
**

**Figure S7. Combinatorial effects of BA145 and autophagy inhibitors on VEGF induced chemotaxis of endothelial cells**. HUVECs at 80-90% confluency, were scratched by pipette and treated with or without 20 ng/mL VEGF along with BA145 (7 µM) and ammonium chloride (10 mM), 3-MA (5mM), LY294002 (10 µM), or bafilomycin (10 nM) for 10 h. Cells were photographed and the migrated cells were quantified by manual counting. Columns, mean; bars, SD; with ***p<0.001, versus BA145 alone.


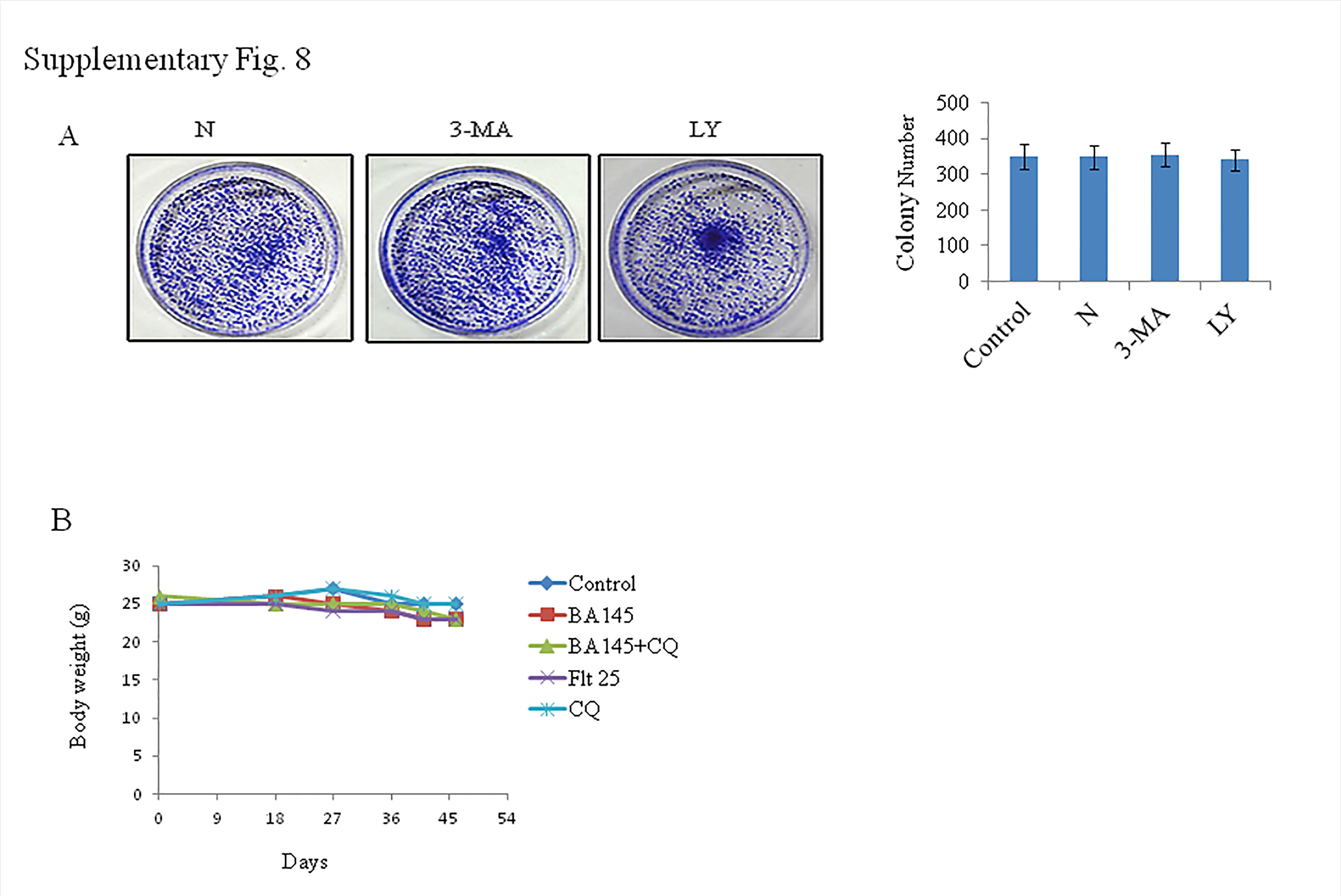


**Figure S8 (A)** Colony formation in ammonium chloride (10 mM), 3-MA (5 mM), or LY294002 (20 µM) treated PC-3 cells after 24 h. Cells were trypsinized and 1000 viable cells were seeded in 60 mm dishes. Cells were allowed to form colonies for 15 days after which colonies were stained with 1% crystal violet and photographed. **(B)** Body weight changes in mice treated with BA145, CQ, and/or flutamide (Flt25). There was no significant difference in body weight between the treated groups and the control group in this study.
